# A Multi-Source Remote-Sensing-Assisted Method for GNSS Station Site Selection in Underground Coal-Mining Subsidence Areas

**DOI:** 10.3390/s26165057

**Published:** 2026-08-09

**Authors:** Yuanrong He, Xiaolin Yu, Huiwei Su, Zhiying Xie, Qun Su, Lisheng Sun, Peiyuan Feng, Zhitian Lin, Ruting Su

**Affiliations:** 1 College of Computer and Information Engineering, Xiamen University of Technology, Xiamen 361024, China; 2012112001@xmut.edu.cn (Y.H.); 2422091020@stu.xmut.edu.cn (X.Y.); 2020000042@xmut.edu.cn (Z.X.); 2422091015@stu.xmut.edu.cn (L.S.); 2422091004@stu.xmut.edu.cn (P.F.);; 2 Guangxi Key Laboratory of Culture and Tourism Intelligent Technology, Guilin Tourism University, Guilin 541006, China; suhuiwei@gltu.edu.cn

**Keywords:** underground coal mining, mining subsidence, GNSS station site selection, InSAR data gaps, multi-source remote sensing, spatial suitability analysis, analytic hierarchy process (AHP), random forest, Gaussian mixture model

## Abstract

Continuous Global Navigation Satellite System (GNSS) monitoring is essential for characterizing surface subsidence in underground coal-mining areas. However, monitoring-station site selection remains strongly dependent on empirical judgment. Furthermore, steep deformation gradients can cause interferometric synthetic aperture radar (InSAR) decorrelation, phase-unwrapping failure, and data gaps in areas where ground-based monitoring is most needed. This study develops a quantitative, multi-source remote-sensing-assisted framework for GNSS monitoring-station site selection under a short-baseline real-time kinematic (RTK) configuration. Sentinel-1A, Sentinel-2C, unmanned aerial vehicle (UAV) photogrammetric products, and road-network data were integrated to construct eight evaluation factors: normalized difference vegetation index (NDVI), slope, terrain ruggedness index, deformation intensity, cumulative subsidence, InSAR coverage, distance to the subsidence edge, and distance to roads. An analytic hierarchy process (AHP) was used as the primary suitability assessment framework, while a random forest (RF) model was introduced with a limited weight to provide auxiliary information only in InSAR data-sparse areas. Grid-level aggregation, three-component Gaussian mixture model (GMM) screening, minimum-distance thinning, field reconnaissance, and monitoring-network review were subsequently combined to convert the continuous suitability surface into deployable station candidates. The resulting high-suitability areas were concentrated mainly along subsidence margins and near InSAR coverage gaps, reflecting the combined effects of deformation representativeness, supplementary monitoring demand, observation conditions, and engineering accessibility. Sixteen candidate stations were identified, of which S12, S3, and S10 were finally recommended to improve the northern, southwestern, and southeastern coverage of the existing monitoring network, respectively. Sensitivity analyses confirmed that the overall suitability pattern and recommended-station rankings remained stable under moderate parameter perturbations. Comparison with two existing GNSS stations further showed that the station with the higher suitability score exhibited a higher fitted subsidence rate (0.438 versus 0.320 mm day^−1^). Given the two-station and two-month validation dataset, this agreement represents preliminary consistency evidence rather than statistical proof of general effectiveness. The proposed framework links regional remote-sensing assessment with site-scale engineering review and monitoring-network optimization, providing practical decision support for GNSS deployment in underground mining-subsidence areas.

## 1. Introduction

In China, coal is predominantly extracted through underground mining, a process associated with high intensity and extensive spatial disturbance [1]. Underground excavation disrupts the in situ stress equilibrium of the overlying strata, triggering overburden movement, surface subsidence, ground fissures, and, in some cases, secondary geohazards. Among these effects, mining-induced subsidence is particularly critical because it threatens buildings, roads, farmland, ecological integrity, and production safety in mining areas [2]. Therefore, continuous and high-precision surface-subsidence monitoring is essential for characterizing subsidence evolution, identifying potential risk zones, and supporting effective mitigation measures.

Among current surface-deformation monitoring techniques, interferometric synthetic aperture radar (InSAR) offers all-weather, wide-area observation with high spatial resolution at relatively low cost, and has become an important technique for mining-induced subsidence monitoring [3]. At the regional scale, InSAR can effectively characterize the spatial distribution of surface deformation and help identify subsidence-basin boundaries and major deformation zones. However, in the core subsidence areas of underground mines, short-term steep deformation gradients often cause phase changes between adjacent pixels to exceed the limit of interferometric processing. This leads to decorrelation, phase-unwrapping failures, and data gaps, preventing reliable measurements in critical deformation zones [3]. Consequently, InSAR alone cannot fully support the detailed and continuous monitoring required in high-risk mining-subsidence areas.

In underground coal-mining subsidence monitoring, such InSAR data gaps should not be interpreted simply as areas with low monitoring demand. When decorrelation or phase-unwrapping failures occur within or adjacent to active subsidence zones, these missing observations may correspond to rapid or large-gradient deformations where continuous ground-based monitoring is most critical. Recent mine-subsidence studies have shown that while InSAR can effectively delineate subsidence basins and their margins, large deformation gradients and decorrelation can make interferometric measurements incomplete or unreliable in central subsidence zones [4]. Consequently, integrated InSAR and GNSS monitoring has been used to improve the completeness and reliability of mining-subsidence interpretation [5].

GNSS monitoring provides continuous, high-temporal-resolution three-dimensional point observations, making it suitable for tracking deformation at key locations in subsidence-sensitive areas, along subsidence margins, and near zones where InSAR observations are incomplete [6]. In engineering practice, mining-subsidence monitoring commonly employs a short-baseline real-time kinematic (RTK) mode, in which monitoring stations receive differential corrections from a relatively stable reference station to generate continuous displacement time series. Accordingly, GNSS monitoring-station site selection is not only an assessment of individual site suitability, but also a network-design problem involving the spatial configuration of the reference station, existing monitoring stations, and newly deployed stations. Stations located far from active subsidence-response zones may fail to capture the dominant deformation process, whereas stations with poor observation conditions, limited accessibility, or high maintenance difficulty may reduce the stability and reliability of long-term monitoring [7,8].

In addition to deformation representativeness and network configuration, GNSS station deployment must satisfy site-specific observational and engineering constraints. Standard GNSS survey specifications require that candidate sites feature an open observation environment, minimal obstructions, sufficient separation from strong radio or electrical interference sources and satellite-signal reflectors, stable foundations, convenient access, and conditions suitable for long-term maintenance [9]. Previous studies on GNSS/SAR infrastructure site-suitability have also emphasized sky visibility, land cover, vegetation obstruction, topography, geological setting, land access, multipath risk, and site security as important criteria for permanent GNSS station deployment [10]. Therefore, an optimal site-selection strategy must holistically integrate deformation representativeness, observational suitability, network configuration, and long-term engineering feasibility.

At present, however, GNSS station site selection in mining areas still relies largely on empirical judgment and localized field reconnaissance. A systematic, quantitative, and reproducible workflow that jointly considers deformation representativeness, InSAR observation gaps, GNSS observation constraints, and engineering feasibility remains underdeveloped [11,12].

Multi-source remote-sensing and spatial datasets provide a basis for the quantitative site selection of GNSS monitoring stations. InSAR-derived deformation fields provide historical deformation information and help identify subsidence-affected zones [13]. UAV photogrammetry can rapidly acquire high-resolution digital elevation models (DEMs) and digital orthophoto maps (DOMs), supporting the extraction of terrain attributes such as slope and terrain ruggedness index (TRI), as well as the interpretation of local surface conditions [14]. The analytic hierarchy process (AHP), a typical multi-criteria decision-making method, can convert expert-based relative-importance judgments into quantitative weights [15]. AHP and related multi-criteria decision-making methods have been widely used in structured decision-making and geographic information system (GIS)-based land-suitability assessment [16,17], and have also been applied in engineering site-selection and GNSS station-suitability studies [12,18,19,20,21].

Most existing studies on GNSS station suitability, however, are tailored to landslide monitoring or general geohazard scenarios, focusing primarily on unstable slopes [11,12]. These assessment frameworks do not translate well to underground coal-mining subsidence monitoring as the monitoring targets, deformation pattern, and specific roles of GNSS observations are different. Mining-induced subsidence is driven by underground extraction and is commonly characterized by concentrated subsidence basins, steep deformation gradients, and fragmented InSAR coverage near subsidence centers [3]. Under such conditions, areas with sparse InSAR coverage should not be automatically excluded from station selection. Instead, their spatial relationship with subsidence-response zones should be evaluated to identify where GNSS observations can most effectively supplement satellite-based monitoring. Furthermore, optimal station placement must extend beyond single-point suitability to account for the spatial relationships among candidate stations, existing reference stations, and existing monitoring stations, thereby avoiding redundant deployment and spatial clustering.

To address these gaps, this study develops a multi-source remote-sensing-assisted framework for GNSS monitoring-station site selection in underground coal-mining subsidence areas. The proposed workflow integrates Sentinel-1A, Sentinel-2C, UAV photogrammetric products, and road-network data to derive eight site-selection factors and generate an initial AHP-based suitability map.

Rather than merely assigning static weights to multiple factors, the workflow is specifically tailored to the unique challenges of mining-subsidence monitoring through four key adaptations: (1) treating InSAR coverage gaps as indicators of supplementary GNSS monitoring demand, (2) using a random forest (RF) model as a constrained auxiliary correction strictly within InSAR data-sparse zones, (3) integrating UAV-derived terrain and orthophoto information for terrain-constraint evaluation and candidate-site review, and (4) converting pixel-scale suitability results into deployable candidate stations through grid-level aggregation, Gaussian mixture model (GMM) screening, minimum-distance thinning, and network-oriented review. In this way, the proposed workflow links regional remote-sensing suitability assessment with practical short-baseline RTK monitoring-station deployment and supports the transformation of pixel-scale suitability results into deployable GNSS monitoring-station candidates.

## 2. Study Area

**Figure 1:**
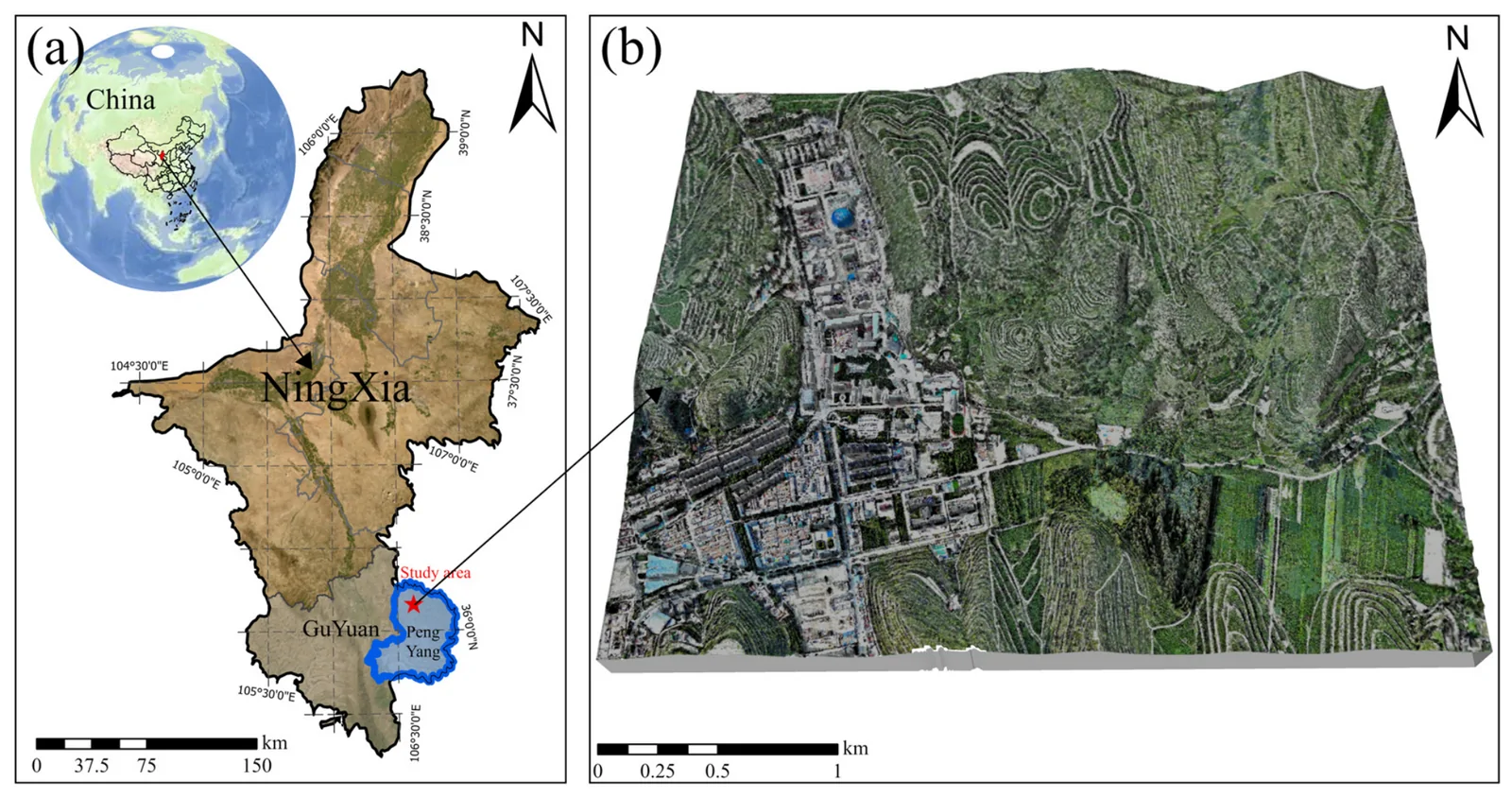
Location and surface characteristics of the study area. (**a**) Geographical location of the study area in Pengyang County, Guyuan City, Ningxia Hui Autonomous Region, China. (**b**) UAV-derived three-dimensional surface view of the study area.

The study focuses on a 4.7 km^2^ subsidence zone within a coal mine in Pengyang County, Guyuan City, Ningxia Hui Autonomous Region, China (Figure 1a). The study area is located in the loess hilly and gully region of the Chinese Loess Plateau. Shaped by Quaternary aeolian loess deposition and long-term fluvial erosion, the terrain is highly fragmented, characterized by dense gullies and undulating ridges, typical of loess hilly landscapes.

The study area has a continental semi-arid climate, with a mean annual temperature of 6–9 °C and an average annual precipitation of 350–430 mm. Precipitation is concentrated between June and September, typically as short, intense rainfall events. Perennial surface runoff is absent, and most gullies serve as seasonal drainage channels.

The mining area is underlain by Paleozoic strata, with the principal coal-bearing formation being the Middle Jurassic Yan’an Formation. This formation consists of continental clastic coal-bearing deposits, with lithologies including grayish-white sandstone, dark-gray siltstone, mudstone, and coal, overlain by thick Cenozoic unconsolidated sediments. The area exhibits moderate structural complexity, with well-developed faults, joints and fractures that affect rock-mass integrity and engineering stability.

Topographic relief and surface conditions exhibit marked spatial heterogeneity across the study area (Figure 1b). The northeastern part is dominated by hilly terrain, where most slopes have been converted to terraces. Farmland is distributed mainly in the central and southern parts, where terraced landforms prevail. In contrast, the western part features gentler terrain, higher residential density, and better road accessibility. This spatial variation in topography, land cover, and accessibility provides a representative setting for GNSS station site selection in mining-subsidence areas.

## 3. Materials and Methods

The GNSS monitoring stations in this study operate in the short-baseline RTK mode. In this mode, monitoring stations are deployed within the subsidence area and receive differential correction data from a reference station located in a relatively stable area. Here, a “relatively stable” area refers to an area outside the primary subsidence-response zone, as identified via InSAR deformation patterns, terrain interpretation, and field reconnaissance, rather than a site proven to be permanently deformation-free. Once fixed RTK solutions are achieved, monitoring stations output three-dimensional displacement components (east, north, vertical), yielding continuous time series. Under favorable observation and communication conditions, millimeter-level precision can be obtained through terminal-based solution processing and filtering. Accordingly, the site-selection problem addressed here focuses on the spatial optimization of GNSS stations for network-based RTK differential monitoring, rather than isolated point selection.

**Figure 2:**
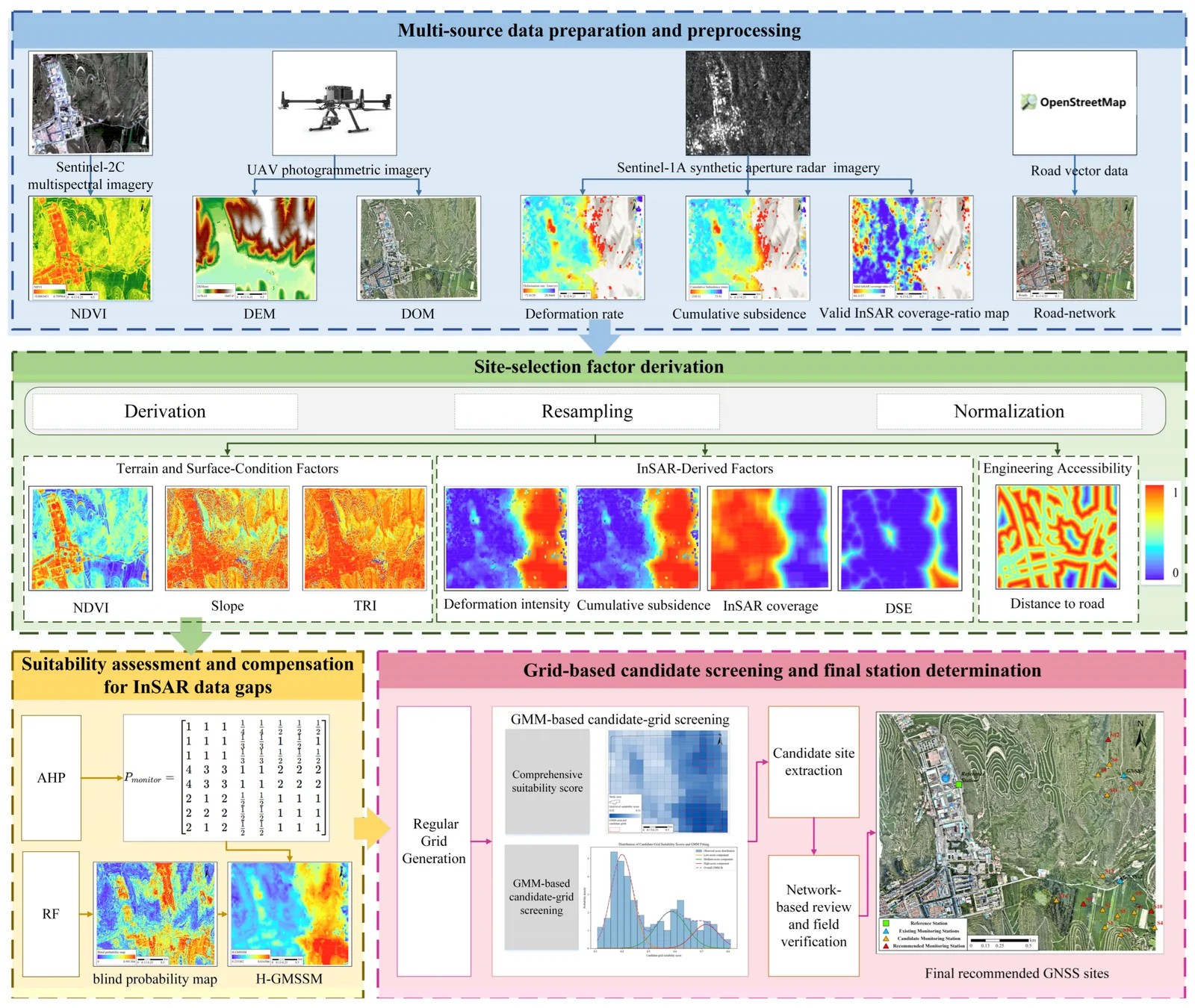
Technical workflow of the proposed GNSS station site-selection framework integrating multi-source remote-sensing data and network-constrained optimization.

Aligned with this RTK monitoring configuration and the operational demands of underground coal-mining subsidence monitoring, this study developed a GNSS station site-selection framework that integrates multi-source remote sensing, spatial filtering, and network-level optimization (Figure 2). The framework considers the surface environment, subsidence characteristics, and construction accessibility of the study area. It includes four steps: multi-source data preprocessing, site-selection factor derivation, suitability assessment with compensation for InSAR data gaps, and grid-based candidate screening and final station determination. The framework aims to quantitatively identify optimal GNSS station locations and provide support for engineering deployment.

### 3.1. Multi-Source Data Preparation and Preprocessing

This study integrated Sentinel-1A synthetic aperture radar (SAR) imagery, Sentinel-2C multispectral imagery, UAV photogrammetric products, and road vector data (Table 1). Sentinel-1A imagery was used to derive historical deformation information and InSAR coverage characteristics in the mining area. Sentinel-2C imagery enabled the extraction of vegetation cover information. UAV-derived DEM and DOM products provided high-resolution terrain attributes and land-cover details. Road vector data quantified accessibility conditions for station installation and maintenance. All datasets were reprojected to World Geodetic System 1984/Universal Transverse Mercator (WGS 84/UTM) Zone 48N, and all site-selection factors were resampled to a spatial resolution of 10 m to ensure spatial consistency across multi-source datasets.

**Figure 3:**
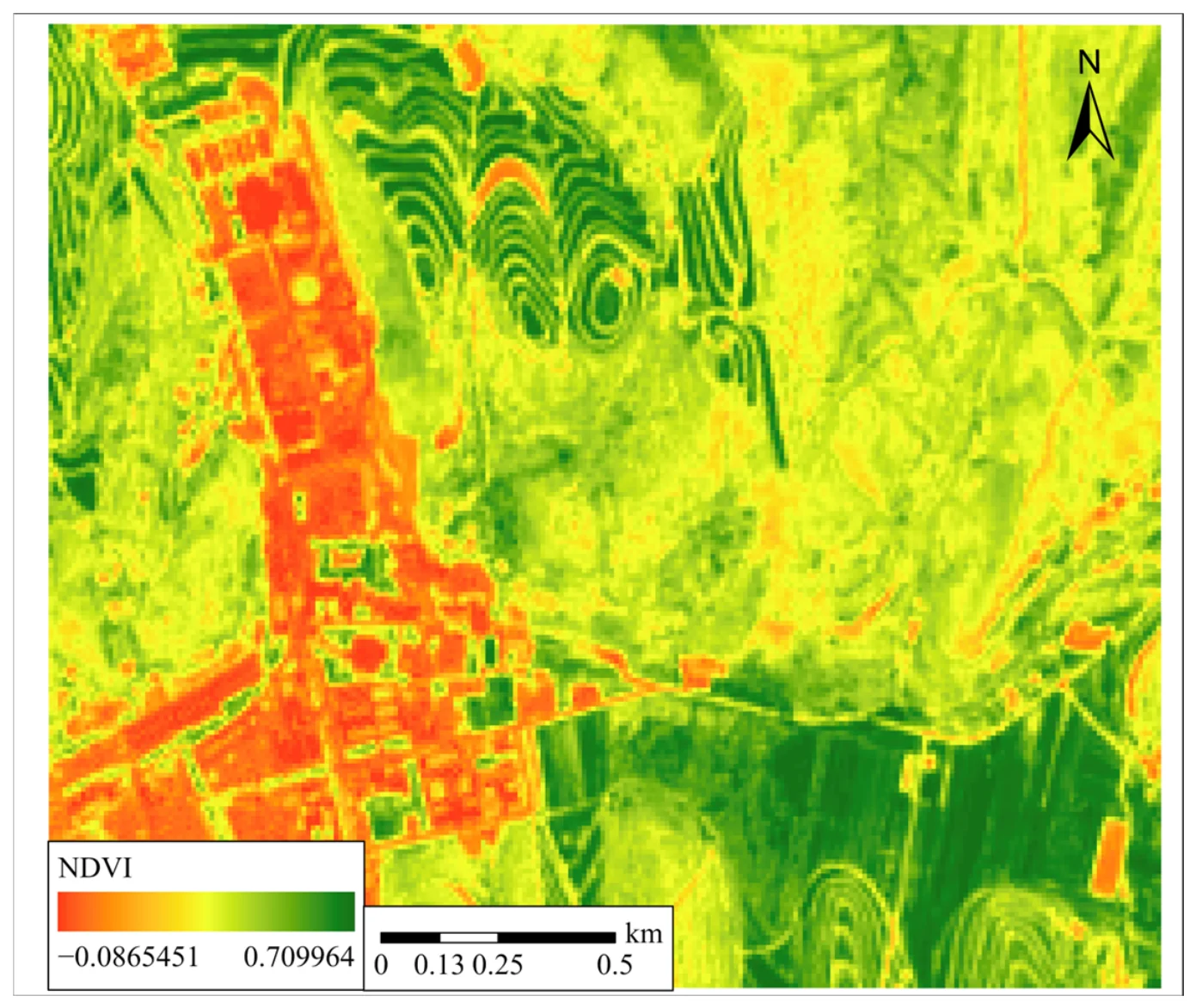
Normalized difference vegetation index (NDVI) map derived from Sentinel-2C imagery (13 August 2025) for vegetation cover assessment.

The Sentinel-2C image acquired on 13 August 2025 was selected to align temporally with the UAV survey, improving the consistency between the extracted vegetation information and the actual land-cover conditions. After preprocessing and format conversion in SNAP 13.0.0, NDVI was calculated in ENVI 6.1 using the near-infrared and red bands [22]. The resulting NDVI map is shown in Figure 3. Higher NDVI values generally indicate denser vegetation, which can obstruct GNSS signals and limit site accessibility for construction and maintenance.

**Figure 4:**
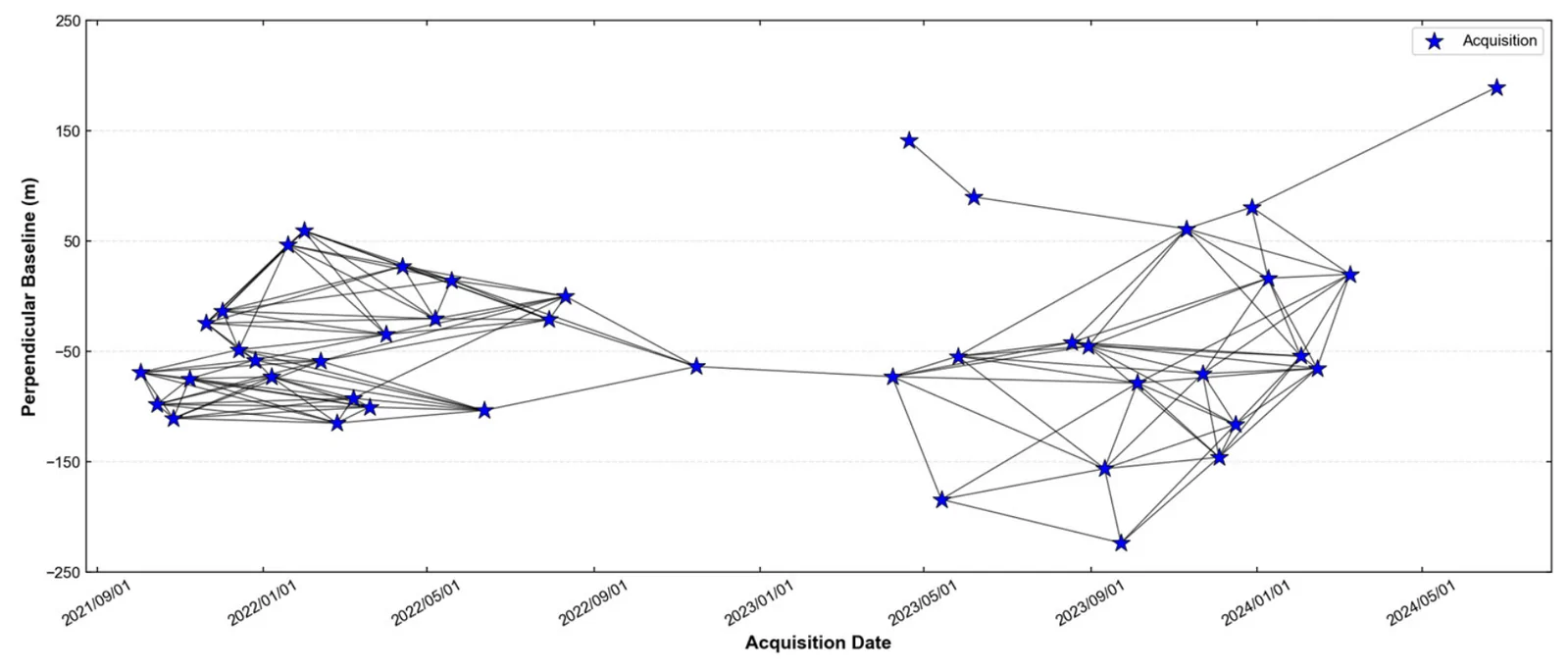
Spatiotemporal baseline network of Sentinel-1A interferometric pairs for SBAS-InSAR time-series analysis (2021–2024).

**Figure 5:**
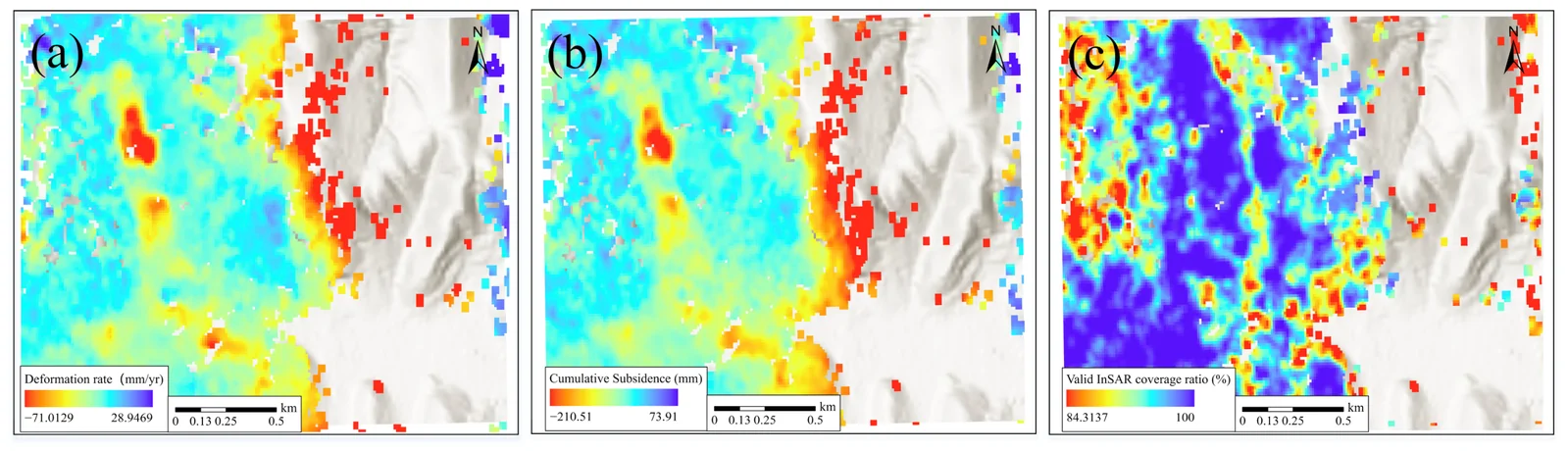
SBAS-InSAR-derived deformation products for the Pengyang mining subsidence area (2021–2024): (**a**) annual mean LOS deformation rate; (**b**) cumulative subsidence; (**c**) valid InSAR coverage ratio. The gray background represents hillshade.

A total of 43 Sentinel-1A SAR images (3 October 2021 to 25 June 2024) were processed using the standard small baseline subset InSAR (SBAS-InSAR) workflow implemented in ENVI SARScape. Spatial and temporal baseline thresholds were set to 150 m and 180 days, producing 153 interferometric pairs. Their spatiotemporal baseline network is shown in Figure 4. During processing, a 30 m DEM was used to remove the topographic phase, Goldstein filtering was applied to improve phase quality, and phase unwrapping was performed using the minimum cost flow algorithm with Delaunay triangulation. The coherence threshold was set to 0.3. The 153 interferometric pairs increased temporal redundancy for time-series inversion, supporting the characterization of deformation evolution along the subsidence-funnel margins. Following time-series inversion, the annual mean line-of-sight (LOS) deformation-rate map, cumulative-subsidence map, and valid InSAR coverage-ratio map were obtained for the study area (Figure 5). However, in the core subsidence zone, C-band temporal decorrelation and steep deformation gradients still caused localized data gaps. Consequently, the SBAS-InSAR products were used not only to characterize valid deformation observations, but also to identify areas where InSAR observations were incomplete and supplementary GNSS monitoring should be considered.

**Figure 6:**
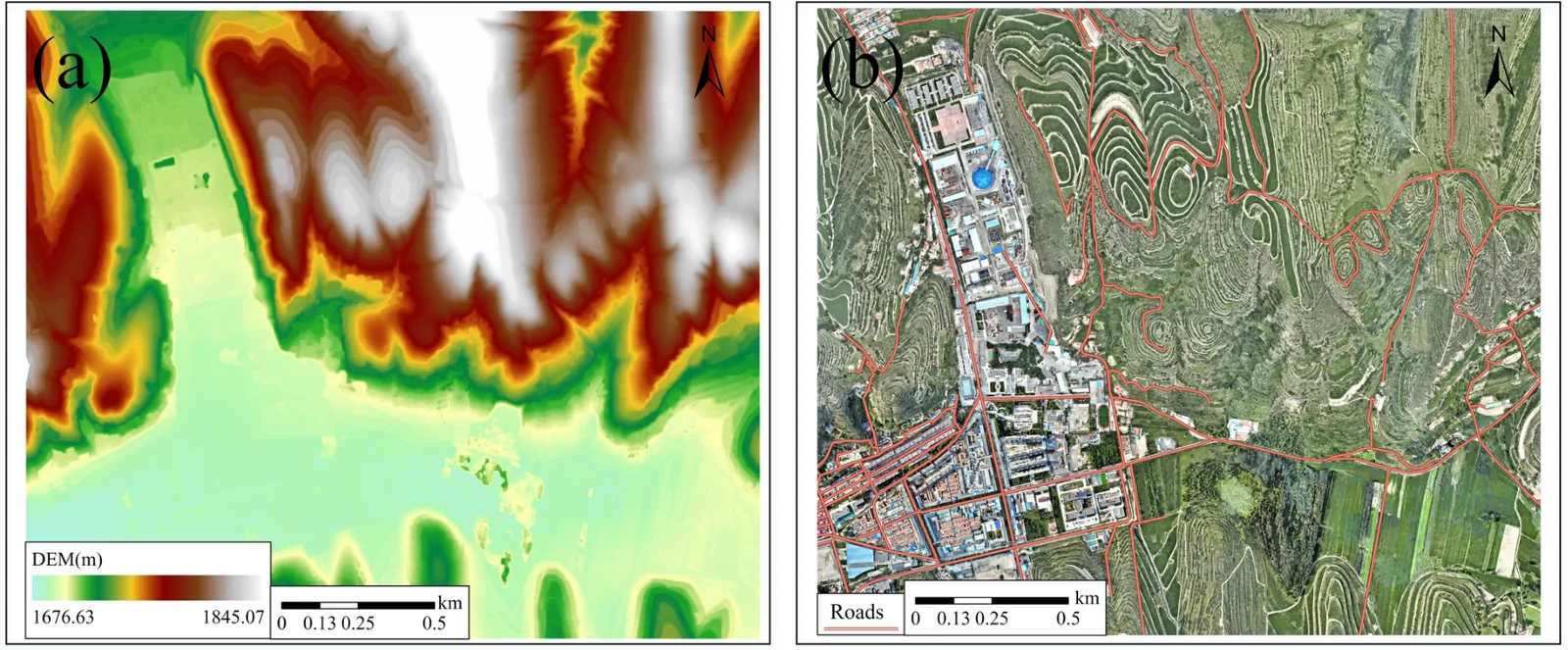
UAV photogrammetric products of the study area: (**a**) digital elevation model (DEM); (**b**) digital orthophoto map (DOM).

**Figure 7:**
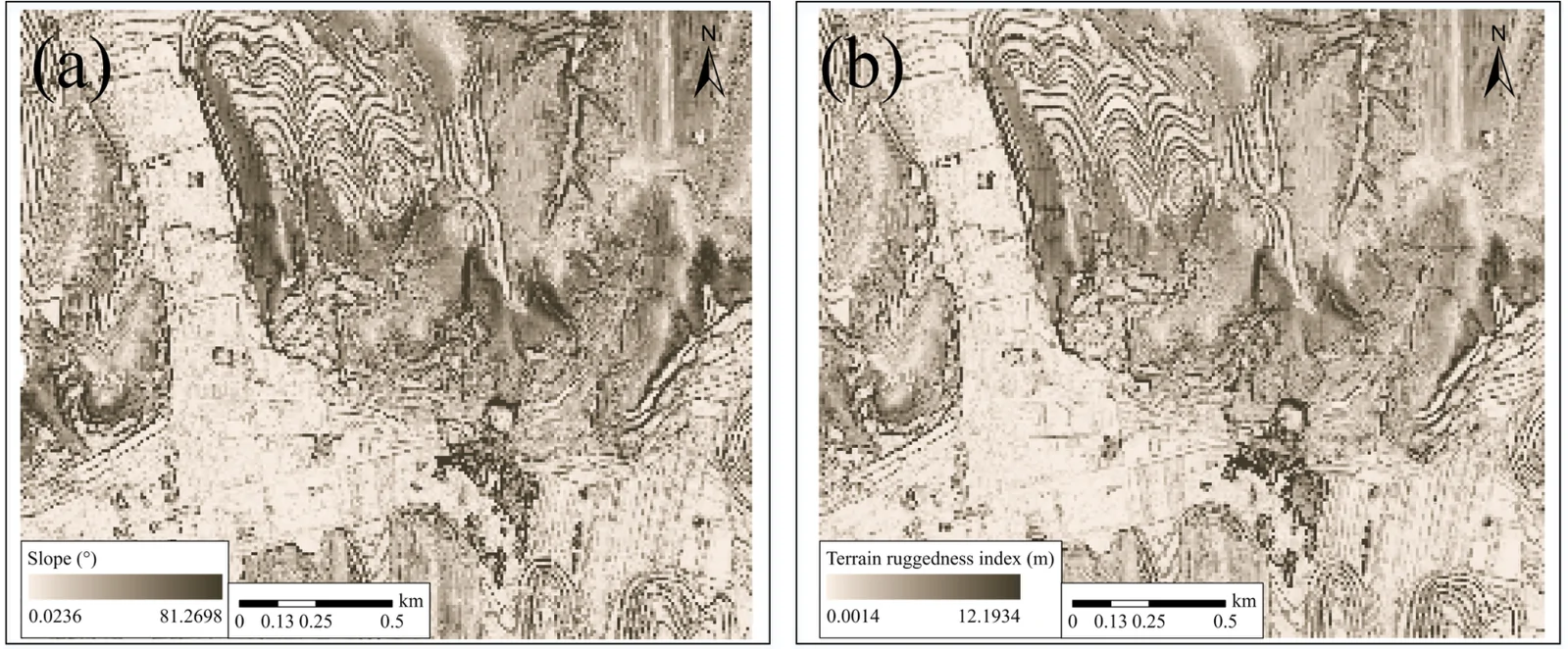
Terrain factors derived from the UAV-derived DEM: (**a**) slope; (**b**) terrain ruggedness index (TRI).

A DJI Mavic 3E UAV acquired the DOM and DEM for the study area (Figure 6). The DOM was used for land-cover visualization, road-network verification, and candidate-site validation. Slope and TRI were calculated from a terrain-filtered UAV-derived DEM, which was generated by removing vegetation and building surfaces from the raw UAV point cloud (Figure 7). Road vector data from OpenStreetMap were overlaid on the UAV-derived DOM for validation and refinement, ensuring accurate representation of current road networks (Figure 6b).

### 3.2. Derivation of Site-Selection Factors

According to the requirements of underground coal-mining subsidence monitoring, eight site-selection factors were derived: NDVI, slope, TRI, deformation intensity, cumulative subsidence, InSAR coverage, distance to subsidence edge (DSE), and distance to road.

#### 3.2.1. Terrain and Surface-Condition Factors

Terrain and surface-condition factors include NDVI, slope, and TRI. NDVI quantifies vegetation-cover density. Higher NDVI values indicate denser vegetation cover, which may reduce local sky openness, increase the risk of signal obstruction, compromise solar-power reliability, and hinder site accessibility. Slope characterizes terrain steepness. Steeper slopes increase the difficulty in constructing stable foundations, installing equipment, and maintaining long-term station integrity. TRI measures local terrain ruggedness. Higher values imply greater effort for site leveling and foundation treatment.

For these three factors, larger original values indicate less favorable conditions for GNSS monitoring-station deployment. Therefore, inverse min–max normalization was used to transform each factor into a suitability value ranging from 0 to 1:

(1) $$S_{i}^{-}=1-\frac{X_{i}-X_{min}}{X_{max}-X_{min}},$$

where *X_i_* is the original value of the *i*-th grid cell, *X*_min_ and *X*_max_ are the minimum and maximum values of the corresponding factor, respectively, and $S_{i}^{-}$ is the normalized suitability value. After normalization, $S_{i}^{-}$ values closer to 1 indicate higher suitability for GNSS station site selection.

#### 3.2.2. InSAR-Derived Factors

Under steep subsidence gradients, InSAR deformation products may not provide spatially continuous and valid observations across the entire study area. Subsidence centers and adjacent zones are particularly susceptible to decorrelation and phase-unwrapping failure, resulting in data gaps [3]. Directly treating these areas as NoData or assigning low suitability scores, risks misclassifying high-deformation zones as low-priority monitoring areas. Therefore, the derivation of InSAR-derived factors in this study considered both valid deformation observations and the need for supplementary GNSS monitoring in data-sparse regions. Four evaluation factors were derived: deformation intensity, cumulative subsidence, InSAR coverage, and DSE.

The InSAR coverage factor was derived from the valid InSAR coverage-ratio raster, which was calculated as the proportion of valid SBAS observations retained per pixel after coherence thresholding, phase unwrapping, and time-series inversion. Within the static analysis mask, pixels lacking observational support (NoData) were assigned a value of 0. A threshold of 60% was then used for binarization: pixels with a coverage ratio ≥ 60% were assigned a value of 1, while the remaining pixels were assigned 0. Sensitivity tests were conducted using coverage-ratio thresholds of 40%, 50%, 60%, 70%, and 80%. The results confirmed that the 60% cutoff serves as a moderate empirical balance, retaining pixels with a majority of valid SBAS observations while avoiding excessive expansion of the data-sparse compensation area. To reduce spatial fragmentation, a focal mean filter was applied to generate a continuous coverage surface, denoted as C_InSAR_. This factor characterizes the spatial continuity and data availability of InSAR observations at and around the target location. A higher value indicates stronger constraints from existing deformation information. As a positive factor, C_InSAR_ was normalized as follows:

(2) $$S_{i}^{+}=\frac{X_{i}-X_{min}}{X_{max}-X_{min}},$$

where *X_i_* is the original value of the *i*-th grid cell, *X*_min_ and *X*_max_ are the minimum and maximum values of the corresponding factor, respectively, and $S_{i}^{+}$ is the normalized suitability value.

To quantify the need for GNSS supplementary monitoring in data-sparse areas, an InSAR supplementary monitoring-demand factor (N_InSAR_) was derived as an intermediate variable:

(3) $$N_{InSAR}=1-C_{InSAR},$$

where a larger *N*_InSAR_ indicates sparser InSAR coverage and a stronger demand for supplementary GNSS observations.

Deformation intensity and cumulative subsidence quantify deformation activity during the monitoring period and long-term cumulative deformation, respectively. Absolute values of the annual mean deformation rate |V| and cumulative subsidence |S_cum_| served as the basic inputs. In regions with valid InSAR observations, the positively normalized |V| and |S_cum_| values were directly used. In data-sparse regions, *N*_InSAR_ was used for gap compensation to avoid the exclusion of areas with missing InSAR data. This process can be expressed as:

(4) $$M_{i}=\left\{\begin{array}{c} m_{i},\;\;\;\;\;\;\;\;\;\;\;m_{i}\neq \mathrm{NoData}\\ N_{InSAR,i},m_{i}=\mathrm{NoData} \end{array}\right.,$$

where *m_i_* denotes the positively normalized value of |V| or |S_cum_|, and *M_i_* represents the gap-compensated suitability score for deformation-intensity or cumulative-subsidence. This operation allows the model to evaluate monitoring value based on actual deformation information in data-reliable areas, while preserving candidate potential in data-sparse areas according to *N*_InSAR_. Both gap-compensated deformation-intensity and cumulative-subsidence factors are bounded within [0, 1], with larger values indicating greater suitability for GNSS station site selection.

The DSE factor quantifies the spatial relationship of candidate locations to the boundary of the subsidence-affected area. Considering that the boundary derived from the original cumulative-subsidence map may be fragmented or discontinuous in the core subsidence zone, we defined areas with |S_cum_| ≥ 20 mm as subsidence-response zones. Data-sparse pixels spatially adjacent to these zones were then incorporated into an auxiliary connection range to improve the continuity of the subsidence boundary. Subsequently, raster-to-polygon conversion, removal of small patches, polygon-to-line conversion, and Euclidean distance calculation were performed to obtain the DSE value for each grid cell.

Zones adjacent to the subsidence edge are commonly located between the subsidence basin and the stable periphery, balancing deformation-monitoring sensitivity with favorable engineering safety. In this study, a Gaussian function was used to derive the DSE suitability score:

(5) $$S_{i}(d_{i})=exp\left(-\frac{{\left(d_{i}-\mu \right)}^{2}}{2\sigma^{2}}\right),$$

where *d_i_* is the Euclidean distance from the *i*-th grid cell to the subsidence edge, *μ* is the optimal distance for GNSS deployment, and *σ* controls the spatial decay rate of suitability.

Considering the spatial scale of the subsidence boundary, station-deployment requirements, field reconnaissance, and site-specific engineering experience, *μ* and *σ* were set to 120 m and 60 m, respectively. This setting indicates that the highest suitability occurs approximately 120 m from the subsidence edge, while relatively high scores are maintained within approximately 60–180 m. The bell-shaped transformation is designed to assign higher suitability to the transitional zone between the active subsidence basin and the relatively stable periphery. Suitability gradually decreases as di deviates from *μ*, reflecting reduced suitability when a location is either too close to the subsidence boundary or too far from the deformation-response zone. To examine the influence of these empirical Gaussian parameters and to compare the selected stations with the theoretical DSE optimum, additional sensitivity tests and station-level distance extractions were conducted. Detailed results are provided in Tables S11 and S12 in the Supplementary Materials.

#### 3.2.3. Distance-to-Road Factor

Beyond meeting the objectives of subsidence monitoring, GNSS station site selection should consider engineering implementation conditions, including material transport, field installation, routine inspection, and long-term maintenance. Based on the preprocessed road vector data, Euclidean distance was calculated from each grid cell to the nearest road to generate the distance-to-road raster. A shorter distance to roads does not necessarily indicate higher suitability. Although locations near roads may facilitate installation and maintenance, they may also be affected by traffic-induced vibrations, human disturbance, and safety constraints. Conversely, excessive distances increase logistical costs and maintenance difficulties. To capture this trade-off, the distance-to-road factor was transformed into an engineering-accessibility suitability score using the Gaussian function defined (Equation (5)).

In accordance with GNSS survey specifications regarding accessibility, long-term point preservation, stable observation conditions, and avoidance of disturbance sources [9], the distance-to-road factor was designed to balance engineering accessibility and road-related disturbance. Based on the road-network distribution, UAV DOM interpretation, and field conditions, *μ* and *σ* were set to 120 m and 90 m, respectively. This parameterization assigns the highest suitability to locations approximately 120 m from the nearest road, while maintaining a relatively broad tolerance range around this preferred distance. The parameter *μ* represents a practical balance between construction and maintenance accessibility and the mitigation of traffic-related disturbance, whereas *σ* controls the tolerance range around this preferred distance. To evaluate the influence of these empirical parameters, road-distance sensitivity tests and selected-station road-distance extraction were conducted, with detailed results provided in Supplementary Tables S11 and S12.

### 3.3. Suitability Assessment and Compensation for InSAR Data Gaps

#### 3.3.1. AHP-Based Suitability Assessment

To integrate multi-source site-selection factors and align with GNSS deployment objectives, AHP was employed to construct a monitoring-station suitability evaluation system. Originally developed by Saaty [15], AHP decomposes a complex decision-making problem into a goal, a criterion, and an indicator layer, and determines relative weights through pairwise comparisons. It is therefore suitable for multi-objective and multi-factor evaluation problems such as GNSS station site selection.

**Figure 8:**
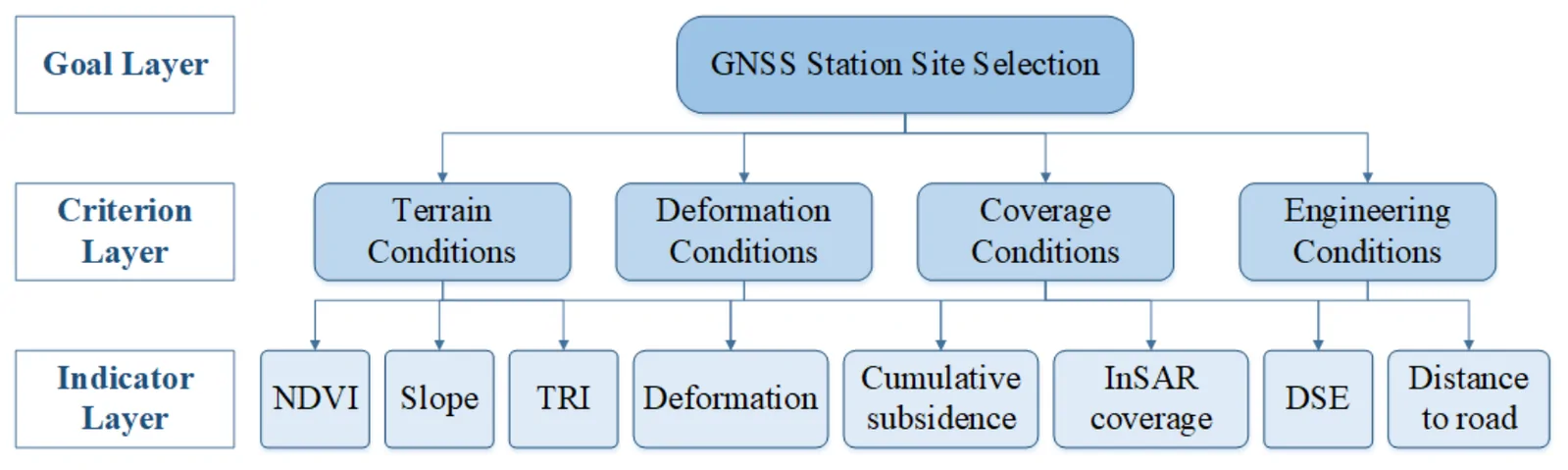
AHP hierarchical structure for GNSS monitoring-station suitability assessment.

The hierarchical structure constructed in this study is shown in Figure 8.

The AHP pairwise comparison matrix was constructed through a project-based expert consultation process. The consultation group comprised four participants: one professor with experience in remote-sensing interpretation and mining-subsidence monitoring, one senior engineer specializing in surveying and GNSS engineering, one project technical manager familiar with the mining area, and one field engineering practitioner responsible for local implementation. The final matrix was formed through group discussion as a consensus judgment matrix for the short-baseline GNSS monitoring-station siting problem.

Pairwise comparisons were conducted according to the relative influence of each evaluation factor on the GNSS station site selection. The resulting judgment matrix P_monitor_ is formulated as follows:

(6) $$P_{monitor}=\left[\begin{array}{cccccccc} 1 & 1 & 1 & 1/4 & 1/4 & 1/2 & 1/2 & 1/2\\ 1 & 1 & 1 & 1/3 & 1/3 & 1 & 1/2 & 1\\ 1 & 1 & 1 & 1/3 & 1/3 & 1/2 & 1/2 & 1/2\\ 4 & 3 & 3 & 1 & 1 & 2 & 2 & 2\\ 4 & 3 & 3 & 1 & 1 & 2 & 2 & 2\\ 2 & 1 & 2 & 1/2 & 1/2 & 1 & 1 & 1\\ 2 & 2 & 2 & 1/2 & 1/2 & 1 & 1 & 1\\ 2 & 1 & 2 & 1/2 & 1/2 & 1 & 1 & 1 \end{array}\right],$$

Judgment matrix elements were assigned based on Saaty’s principle of relative-importance [15]. Rather than using the full 1–9 Saaty scale, this study adopted a truncated 1–4 scale and its reciprocals. The truncated scale was used because the eight factors were considered to have moderate rather than extreme differences in relative importance under the short-baseline GNSS monitoring context. This conservative setting reduces the risk of over-amplifying subjective judgments and prevents any single factor from becoming excessively dominant in the final suitability model. The scale definitions are listed in Table 2.

The consistency of the AHP judgment matrix was evaluated using the consistency index CI and consistency ratio CR:

(7) $$CI=\frac{\lambda_{max}-n}{n-1},$$

(8) $$\mathrm{CR}=\frac{\mathrm{CI}}{\mathrm{RI}},$$

where *λ*_max_ is the maximum eigenvalue of the judgment matrix, *n* is the matrix order, and *RI* is the random consistency index. When *CR* < 0.10, the judgment matrix satisfies the consistency requirement. In this study, the order of the judgment matrix was 8, corresponding to *RI* = 1.41. The consistency test showed that *λ*_max_ = 8.0717, *CI* = 0.0102, and *CR* = 0.0073, satisfying the requirement of *CR* < 0.10.

The AHP-derived weights for NDVI, slope, TRI, deformation intensity, cumulative subsidence, InSAR coverage, DSE, and distance to road were 0.0607, 0.0792, 0.0654, 0.2250, 0.2250, 0.1116, 0.1214, and 0.1116, respectively. Deformation intensity and cumulative subsidence jointly account for 45% of the total weight, reflecting the model’s prioritization of subsidence-responsive zones. The remaining weights balance InSAR observation constraints, subsidence-edge monitoring requirements, and engineering feasibility. To examine whether the final suitability pattern was dominated by subjective uncertainty in the AHP weights, an AHP weight sensitivity analysis was further conducted. Both individual and grouped factor weights were independently perturbed by ±20%, with the remaining weights proportionally renormalized. The normalized factor rasters were kept constant to strictly isolate the effect of AHP weight variation. Raster-level similarity, top-10% high-suitability overlap, and selected-station rank stability were recalculated under each perturbation scenario. Detailed results are provided in Tables S7 and S8 in the Supplementary Materials.

Based on the standardized evaluation factors and AHP weights, the GNSS monitoring-station suitability map (GMSSM) was generated using weighted linear combination:

(9) $$GMSSM_{i}=\sum_{j=1}^{8}w_{j}S_{ij},$$

where GMSSM*_i_* is the GNSS monitoring-station suitability value of the *i*-th grid cell, *w*_*j*_ is the AHP weight of the *j*-th evaluation factor, and *S*_*ij*_ is the standardized suitability value of the *i*-th grid cell for the *j*-th evaluation factor.

#### 3.3.2. RF-Assisted Data-Sparse Zone Correction

To differentiate deformation potential across locations within InSAR data-sparse zones, an RF model was introduced for auxiliary identification [23]. The input features of the RF model were NDVI, slope, and TRI. These variables were selected as spatially continuous descriptors of surface conditions that govern how mining-induced deformation is expressed, observed, and practically monitored at the ground surface. NDVI characterizes vegetation cover and land-surface conditions, which can affect the visibility of surface damage, the continuity of InSAR observations, and the feasibility of GNSS station installation. Slope and TRI describe terrain gradient and local relief. In loess hilly and gully mining areas, mining-induced subsidence may interact with slope instability and gully-side deformation. Therefore, terrain gradient and roughness can modulate the surface expression of subsidence-related deformation. Previous studies in mountainous mining areas have shown that sparse vegetation, large slopes, and slope instability can influence the expansion of mining-affected areas and the interpretation of SBAS-InSAR-derived subsidence boundaries [24]. Accordingly, the RF model was trained to learn the empirical association between these surface-condition variables and the integrated deformation response D in InSAR-valid training areas, and to provide an auxiliary indication of potential high deformation response in InSAR data-sparse zones. The response variable was constructed by equally combining the original positively normalized deformation-intensity and cumulative-subsidence factors:

(10) $$D_{i}=0.5M_{V,i}^{raw}+0.5M_{S,i}^{raw},$$

where $M_{V,i}^{raw}$ and $M_{S,i}^{raw}$ denote the original positively normalized deformation-intensity factor and cumulative-subsidence factor of the *i*-th grid cell, respectively. This equal-weighted combination considers both deformation activity during the monitoring period and the long-term cumulative deformation level, thereby reducing the bias that may arise from using a single indicator to represent subsidence sensitivity.

Training samples for the RF model were restricted to pixels with an InSAR coverage ratio ≥ 0.6, ensuring that the training labels were derived from areas with adequate InSAR observational support. The upper quartile of the integrated response *D* was used as the positive-sample threshold. Grid cells satisfying $D_{i}\geq Q_{75}(D)$ were labeled as potential high-deformation-sensitivity areas, whereas the remaining samples were labeled as non-high-sensitivity areas:

(11) $$y_{i}=\left\{\begin{array}{c} 1,D_{i}\geq Q_{75}(D),\\ 0,{\;D}_{i}<Q_{75}(D), \end{array}\right.$$

where *y_i_* is the training label of the RF model, and *Q*_75_(*D*) represents the upper quartile of the integrated response *D*. This threshold was used to emphasize the discriminative characteristics of areas with relatively strong subsidence activity. Because *Q*_75_(*D*) represents an empirical definition of relatively high deformation response rather than an independently verified deformation class, two alternative thresholds, *Q*_70_(*D*) and *Q*_80_(*D*), were tested together with the baseline *Q*_75_(*D*). This threshold-sensitivity test was used to evaluate whether the RF-assisted correction was strongly dependent on the positive-label definition.

Key RF hyperparameters were set as follows: 300 decision trees, a minimum leaf size of 5, balanced subsample class weights, and a fixed random seed of 42. For each positive-label threshold, the RF model was evaluated using a stratified 70/30 train–test split, with performance assessed via out-of-bag (OOB) error, area under the receiver operating characteristic curve (AUC), precision, recall, F1-score, confusion matrix, and feature importance ranking. To justify the selection of RF as the appropriate auxiliary classifier, Logistic Regression, AdaBoost, Gradient Boosting, and XGBoost were also trained using the same input features and the *Q*_75_(*D*) labels. The detailed RF threshold-sensitivity results and classifier-comparison results are reported in Supplementary Tables S1 and S2, respectively.

To integrate the AHP-derived suitability map with the RF-based auxiliary probability layer, a zonal fusion strategy was adopted. In InSAR data-sparse areas, the RF-derived probability $P_{blind\;}$ was introduced with a constrained weight to supplement the AHP-based suitability value. In areas with adequate InSAR coverage, the original AHP-derived suitability value was retained:

(12) $${GMSSM}_{i}^{hybrid}=\left\{\begin{array}{c} 0.85{GMSSM}_{i}+0.15\times P_{blind,i\;},\;\;\;C_{InSAR,i}<0.6\\ {GMSSM}_{i}\;,\;\;\;\;\;\;\;\;\;\;\;\;\;\;\;\;\;\;\;\;\;\;\;\;\;\;\;\;\;\;\;\;\;\;\;\;\;C_{InSAR,i}\geq 0.6 \end{array}\right.,$$

where ${GMSSM}_{i}^{hybrid}$ is the suitability value of the *i*-th grid cell after RF-assisted compensation, GMSSM*_i_* is the original AHP-derived suitability value, $P_{blind,i\;}$ is the RF-derived probability of potential high deformation response, and $C_{InSAR,i}$ is the InSAR coverage value.

The RF weight was set to 0.15 to maintain the AHP-derived GMSSM as the primary suitability assessment framework while allowing a conservative local adjustment in InSAR data-sparse zones. To evaluate the influence of this empirical setting, RF weights of 0, 0.05, 0.15, and 0.25 were tested, where the weight of 0 represents the AHP-only case without RF compensation. Using the 0.15 case as the reference, the alternative weighting scenarios showed high raster-level similarity and stable candidate-station rankings. This confirms that the RF-derived probability layer was used as a constrained auxiliary component rather than a dominant factor in shaping the final suitability pattern. Detailed raster-level and candidate-level sensitivity results are provided in Supplementary Tables S3 and S4.

### 3.4. Grid-Based Candidate Screening and Final Station Determination

To mitigate the impact of spatial outliers and reduce excessive clustering of candidate sites, the fused suitability map was first smoothed using a focal mean filter. Subsequently, a 150 m × 150 m regular grid was overlaid across the study area. The 150 m grid size was selected as a practical aggregation scale because it exceeds both the 10 m factor resolution and the minimum station-separation distance, while still preserving local spatial differences within the subsidence-margin zone. Grid-based processing allows suitability into a spatial scale more consistent with practical site-selection decision-making, thereby reducing redundant deployment caused by localized clusters of high-value pixels. To evaluate the influence of this empirical aggregation scale, additional grid-size sensitivity tests were conducted using alternative grid sizes of 100 m, 150 m, and 200 m. Detailed results are provided in Supplementary Table S9.

Based on the grid units, zonal statistics were used to extract the maximum and mean suitability values within each grid. A comprehensive grid score was then constructed as follows:

(13) $$G_{k}=0.7{MAX}_{k}+0.3{MEAN}_{k},$$

where *G_k_* is the comprehensive suitability score of the *k*-th grid, MAX*_k_* is the maximum suitability value within the grid, and MEAN*_k_* is the mean suitability value of the grid. The 0.7/0.3 weighting scheme gives priority to the best local pixel for deployment while reducing the influence of isolated high-value spatial outliers. To evaluate the influence of this empirical weighting scheme, additional tests were conducted using alternative MAX/MEAN weighting ratios of 0.5/0.5, 0.7/0.3, and 0.9/0.1, with detailed results provided in Supplementary Table S9.

**Figure 9:**
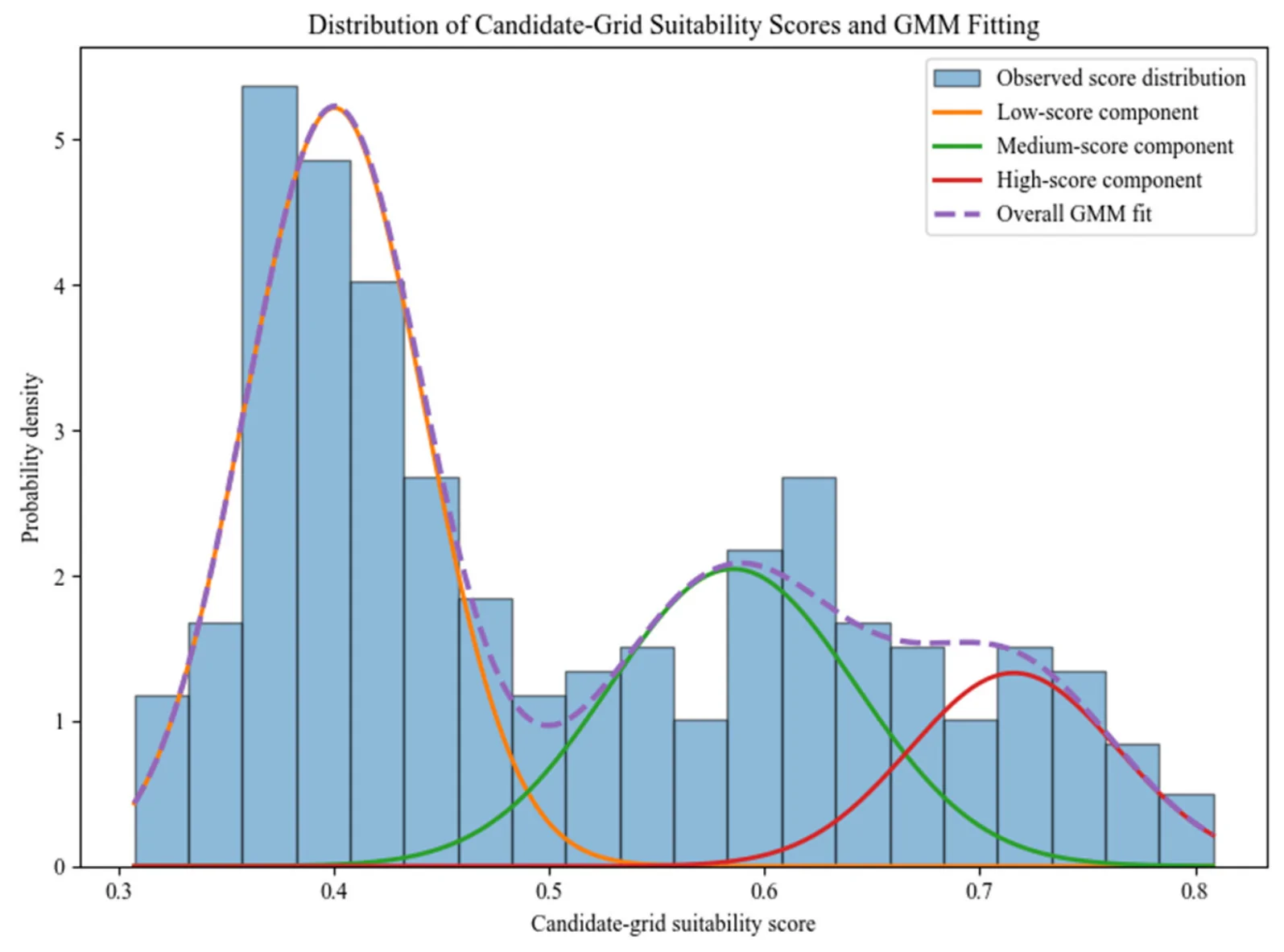
Distribution of candidate grid suitability scores and the fitted three-component Gaussian mixture model (GMM).

After computing the comprehensive grid scores, a GMM was used for probabilistic candidate-grid screening [25]. To determine an appropriate number of components, GMMs with one to six components were fitted to the candidate-grid scores, and their Akaike information criterion (AIC) and Bayesian information criterion (BIC) values were compared. Although the two-component model produced the lowest AIC and BIC values, it merely separated the grids into broad low- and high-suitability categories, retaining 107 high-suitability grids (44.96% of all valid grids). This retained area was too broad to extract a manageable set of practical GNSS candidate grids. In contrast, the three-component model divided the grid-score distribution into low-, medium-, and high-suitability groups, with component means of 0.400, 0.585, and 0.716, respectively. The high-suitability component retained 34 grids, accounting for 14.29% of all valid grids. Therefore, considering both screening selectivity and interpretability, the three-component GMM was used for candidate-grid extraction. A grid was retained as a high-potential candidate grid only when it was assigned to the highest-mean component and its posterior probability for that component was at least 0.60. Detailed AIC/BIC and grid-retention results are provided in Supplementary Table S13, and the fitted GMM distribution is shown in Figure 9.

For each retained high-suitability grid, the pixel with the highest suitability value was selected as the representative candidate point. When multiple pixels had the same maximum suitability value, the pixel closest to the grid center was selected to reduce boundary-position uncertainty.

To reduce local spatial redundancy among candidate points, a minimum-distance thinning procedure was applied following the GMM-based candidate-grid extraction. The candidate points were first sorted in descending order of their suitability scores and then retained sequentially. A candidate was retained only if its Euclidean distance to all previously retained candidates exceeded the specified threshold; otherwise, it was removed as a redundant point within the same local high-suitability patch. In the baseline setting, the minimum-distance threshold was set to 120 m. This threshold was selected as a practical redundancy-control distance because it is larger than the 10 m raster resolution and can reduce repeated representation of the same local suitability patch, while avoiding excessive removal of potentially complementary candidate points. To evaluate the influence of this empirical threshold, additional tests were conducted using alternative thresholds of 80, 100, 120, 150, and 200 m. Detailed results are provided in Supplementary Table S10. Under the baseline 120 m setting, 16 candidate points were retained for subsequent network-level and field-based review.

After minimum-distance thinning, the retained candidate points underwent further monitoring-network review and field reconnaissance. The review considered the spatial complementarity of the candidate points relative to the existing reference and monitoring stations, redundancy within the same local subsidence-response zone, and preliminary site-scale GNSS and engineering conditions. During field reconnaissance, local sky openness, nearby potential signal reflectors, surface-foundation conditions, construction safety, and related factors were qualitatively screened. These checks served as preliminary field-screening constraints. Detailed satellite geometry, DOP, receiver-level signal quality, quantitative radio-frequency interference assessment, and fixed-solution performance remained pre-construction verification items. In addition, given the short-baseline RTK configuration of the monitoring system, the baseline distance from each candidate point to the existing reference station was evaluated according to the cited GNSS survey specification [9]. Because these conditions cannot be fully characterized using regional remote-sensing data layers, they were applied directly as constraints during the final field-screening and monitoring-network review stages.

Based on the retained candidate points, the final station recommendation was determined through a network-oriented review. Candidate points close to existing monitoring stations or representing the same local subsidence-response zone were deprioritized to reduce monitoring redundancy. Preference was given to candidates that improved spatial complementarity and covered subsidence-response zones not sufficiently represented by the existing stations. The final decision jointly considered candidate suitability score, monitoring-network complementarity, field-reconnaissance evidence, construction feasibility, and long-term maintenance conditions.

## 4. Results and Validation

### 4.1. Results

#### 4.1.1. Spatial Distribution of Evaluation Factors and Suitability Scores

**Figure 10:**
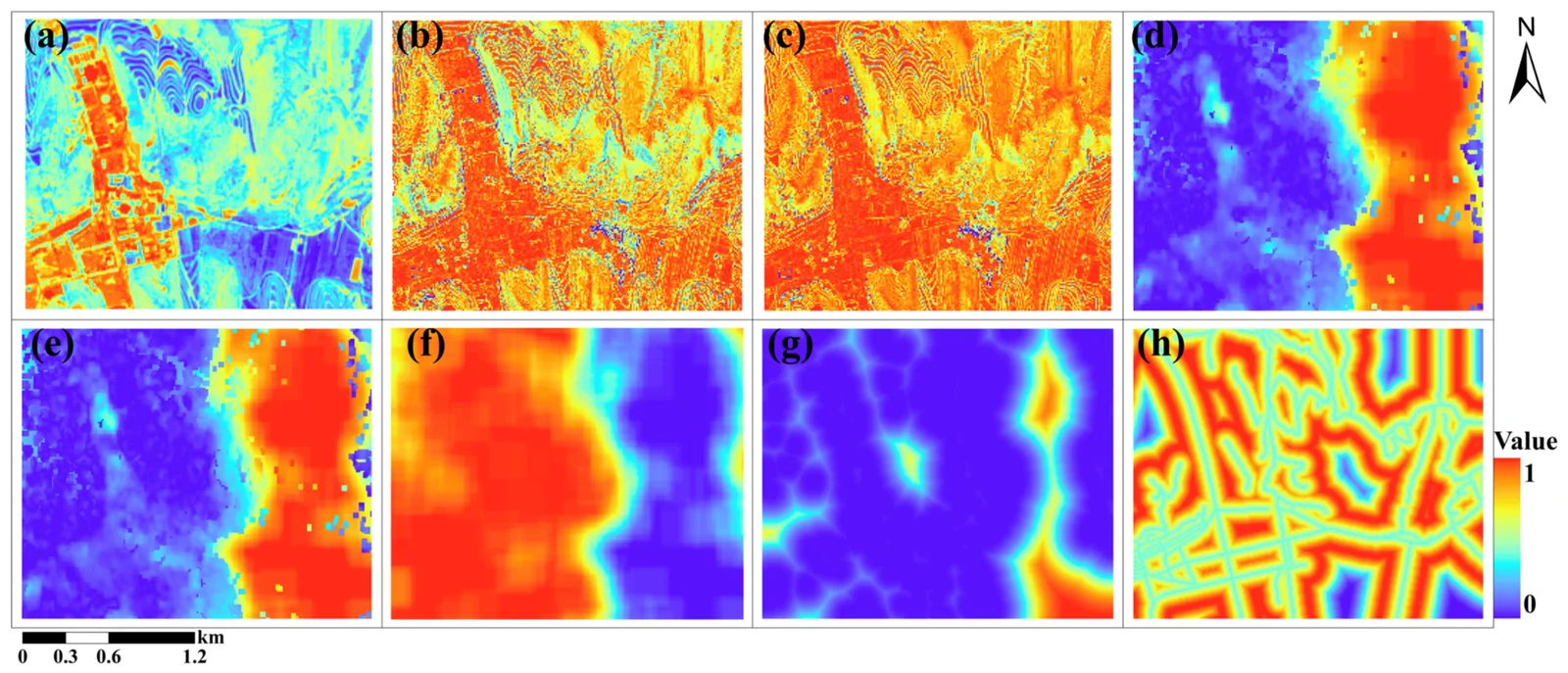
Spatial distribution of the evaluation factors for GNSS station site selection: (**a**) NDVI; (**b**) slope; (**c**) TRI; (**d**) deformation intensity; (**e**) cumulative subsidence; (**f**) InSAR coverage; (**g**) DSE; and (**h**) distance to road.

The eight evaluation factors showed clear spatial heterogeneity across the study area, reflecting differences in land cover, terrain attributes, subsidence response, InSAR observability, and engineering accessibility. The NDVI-derived suitability was mainly controlled by land-cover types (Figure 10a). Lower scores corresponded to farmland and densely vegetated areas, whereas higher scores were observed in bare soil, road-adjacent areas, and sparsely vegetated areas. The slope and TRI factors were mainly controlled by terrain relief (Figure 10b,c). Higher scores were concentrated in flat, construction-friendly areas, whereas lower values occurred on hilly slopes, deeply incised gullies, and in locally rugged terrain.

For the subsidence-response factors, deformation intensity and cumulative subsidence showed broadly similar spatial patterns (Figure 10d,e). High-value areas were mainly distributed within the subsidence basin and its transitional margins, reflecting the active subsidence center and boundary-gradient characteristics of the study area. InSAR coverage showed reduced values or localized data gaps in the core area of steep-gradient subsidence, indicating strong effects of decorrelation and phase-unwrapping failure (Figure 10f). In contrast, InSAR observations maintained higher spatial continuity along subsidence margins and surrounding areas. The DSE factor formed a continuous medium- to high-value belt near the subsidence boundary, representing the spatial relationship between the basin periphery and the active deformation core (Figure 10g). The distance-to-road factor showed relatively higher suitability within a reasonable distance from roads, whereas some remote areas remained less favorable because of limited construction and maintenance accessibility (Figure 10h).

**Figure 11:**
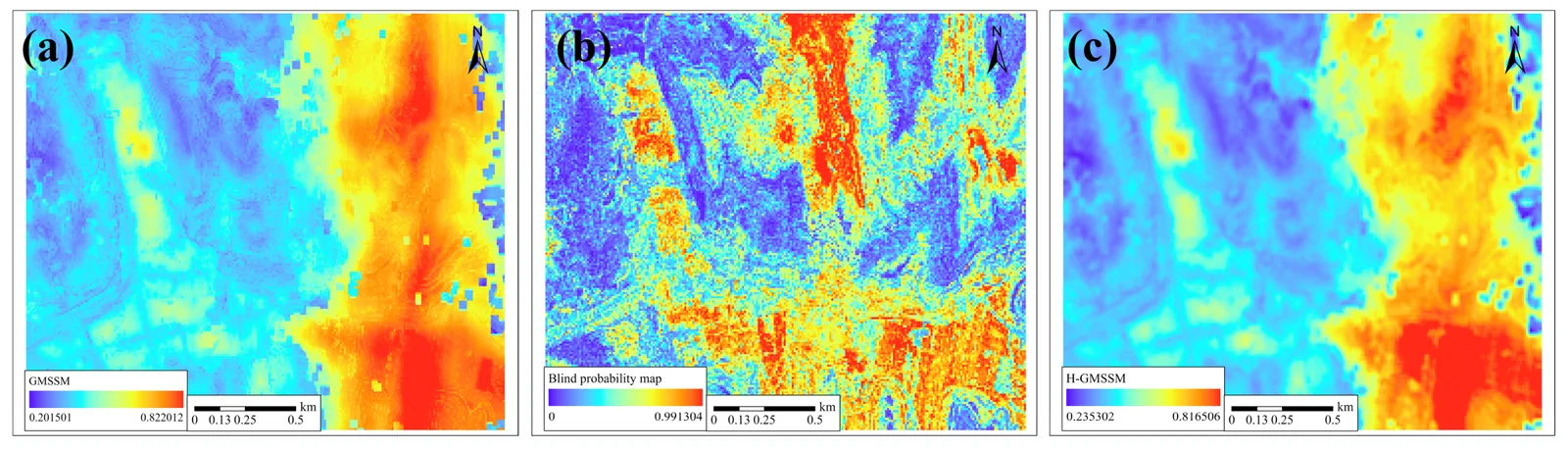
Suitability assessment results: (**a**) GMSSM; (**b**) RF-derived high-deformation-response probability layer for InSAR data-sparse zones; and (**c**) H-GMSSM.

The GMSSM generated from the eight evaluation factors and the AHP weights indicates pronounced spatial heterogeneity in suitability for GNSS station site selection across the study area (Figure 11a). High-suitability areas cluster in areas with active subsidence, proximity to the subsidence edge, and moderate road accessibility. Low-suitability areas are mostly distributed far from key subsidence-response zones or in areas with poor engineering conditions. Overall, the GMSSM effectively reflects the combined influence of subsidence-response capacity, InSAR observation constraints, and engineering feasibility.

The RF-derived deformation-sensitivity probability map for the data-sparse zone shows that high-probability areas are mainly distributed near the subsidence center and along segments of the InSAR coverage-gap margins, exhibiting good spatial adjacency with active subsidence zones (Figure 11b). The fused GNSS monitoring-station suitability map (H-GMSSM) preserves the primary spatial structure of the GMSSM while enhancing suitability continuity in and around data-sparse regions. The suitability of some locations adjacent to data-sparse zones is moderately enhanced (Figure 11c).

#### 4.1.2. Candidate-Grid Screening and Final Station Selection

**Figure 12:**
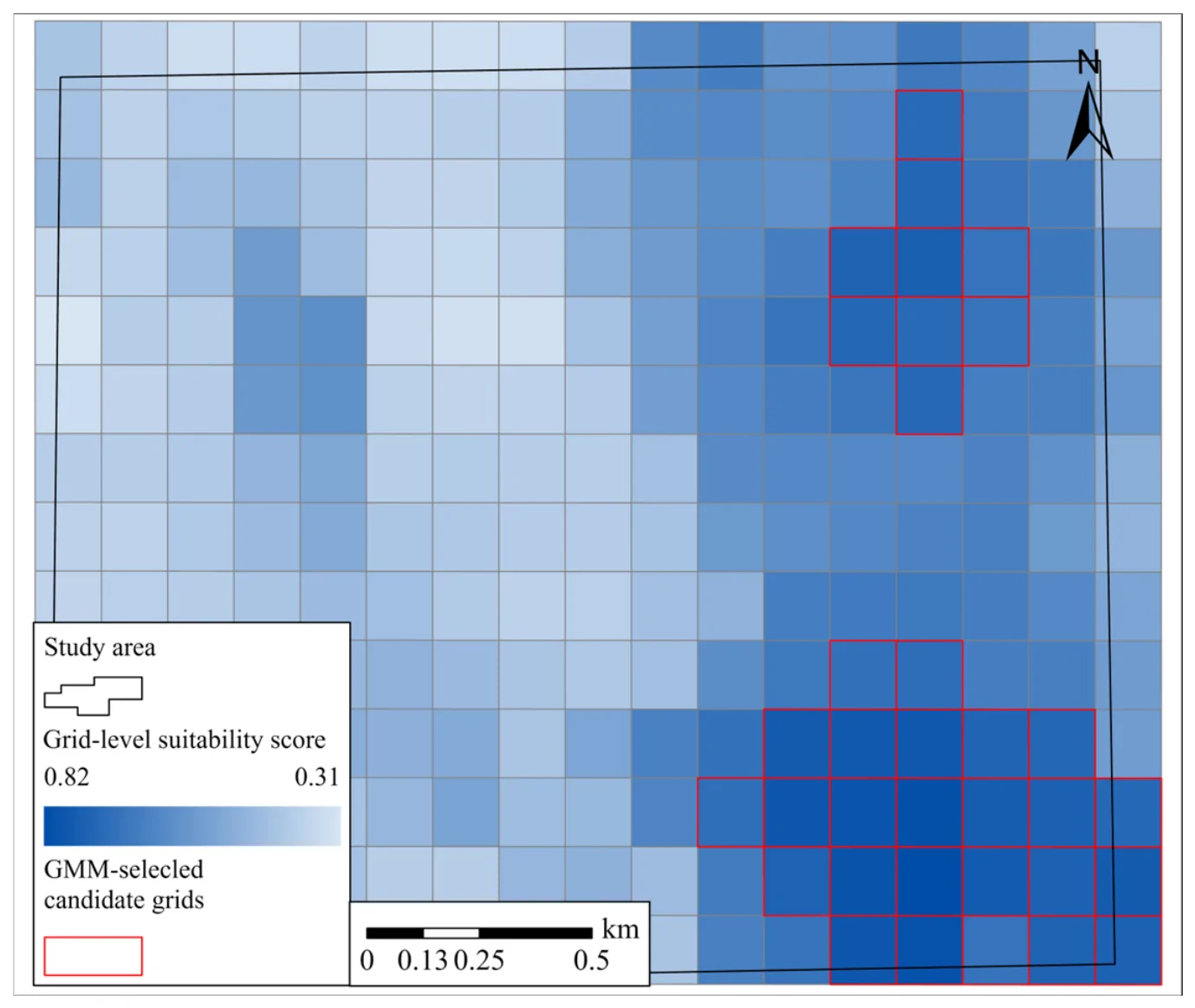
High-potential candidate grids identified by three-component GMM screening.

For each grid unit, the maximum, mean, and comprehensive suitability scores were calculated from H-GMSSM. Compared with the pixel-scale suitability map, the comprehensive grid score reduces the influence of spatial outliers and emphasizes both the overall suitability of candidate areas and their internal optimal deployment potential. As shown in Figure 12, most grid units exhibit medium to low scores, whereas only a limited number of grids exhibit high comprehensive suitability. These high-score grids are mainly distributed along the subsidence margins and in areas adjacent to low-InSAR-coverage zones, indicating that high-potential monitoring areas are spatially concentrated.

The three-component GMM was applied to screen spatially continuous high-suitability areas, yielding a discrete set of high-potential candidate grids (Figure 12). The retained grids cluster in areas with pronounced subsidence, well-defined subsidence margins, and moderate road accessibility. The GMM-based screening replaces a single fixed suitability cutoff with distribution-based grouping and improves the interpretability of candidate-grid extraction. After extracting the optimal pixel within each retained grid and applying the minimum-distance constraint, 16 GNSS candidate stations were obtained and assigned fixed identifiers, S1–S16, for subsequent monitoring-network review.

During the monitoring-network review stage, the 16 candidate points were evaluated together with the existing reference station and two existing GNSS monitoring stations. In addition to evaluating spatial complementarity and redundancy relative to the existing monitoring network, field reconnaissance was used to qualitatively screen preliminary site-scale deployment conditions. The screening considered local sky openness, nearby potential signal reflectors, surface-foundation conditions, construction safety, and related factors. Before station construction, detailed verifications of satellite geometry, DOP, receiver-level signal quality, quantitative radio-frequency interference, and fixed-solution performance should be conducted.

Candidates S6, S16, S11, and S13 were excluded because they were spatially close to existing monitoring stations or located within the same local subsidence-response zone, which would increase monitoring redundancy. Field reconnaissance indicated that S12 was located on relatively flat and open terrain with favorable access conditions, making it suitable for supplementing the northern part of the monitoring network. In the southwestern part of the study area, S14 was rejected because dense vegetation and local obstruction could compromise sky-view conditions and solar-power reliability. In contrast, S3 was selected as a practical alternative for monitoring the southern deformation-response zone and the adjacent InSAR coverage gap. In the southeastern part of the study area, S10 was prioritized over S4 because it had more favorable accessibility and construction–maintenance conditions.

The remaining candidates, including S1, S2, S5, S7, and S9, were subsequently excluded to reduce redundancy with the selected S3 or S10 stations. S15 was excluded despite its southern location, because it lies on the midslope of terraced hills, resulting in relatively poor road accessibility and maintenance conditions. Finally, S12, S3, and S10 were selected as new GNSS monitoring-station locations, corresponding to the northern, southwestern, and southeastern candidate areas, respectively.

Because the final recommended stations were intended to operate within a short-baseline RTK monitoring network, their baseline distances to the existing reference station were further checked together with the two existing monitoring stations. The baseline lengths from the existing reference station to GNSS-1, GNSS-2, S3, S10, and S12 were 1.46 km, 1.65 km, 1.50 km, 2.01 km, and 1.38 km, respectively. According to the baseline precision model specified in the cited GNSS survey specification [9], the baseline measurement standard error can be estimated as:

(14) $$\sigma_{d}=\sqrt{a^{2}+(b\cdot d{)}^{2}}$$

where *a* is the fixed error, *b* is the proportional error coefficient, and *d* is the baseline length. Using the D-grade GNSS parameters *a* ≤ 10 mm and *b* ≤ 10 mm/km, the estimated baseline standard errors for GNSS-1, GNSS-2, S3, S10, and S12 were 17.7 mm, 19.3 mm, 18.0 mm, 22.5 mm, and 17.0 mm, respectively. These values represent the expected standard-error scale derived from the D-grade GNSS baseline precision model [9]. Within the relatively narrow baseline range of 1.38–2.01 km, baseline length was therefore treated as a feasibility reference rather than as the principal factor for candidate ranking, while actual RTK positioning accuracy still requires verification through field observations and operational quality assessment.

### 4.2. Small-Sample Validation Using Existing GNSS Stations

**Figure 13:**
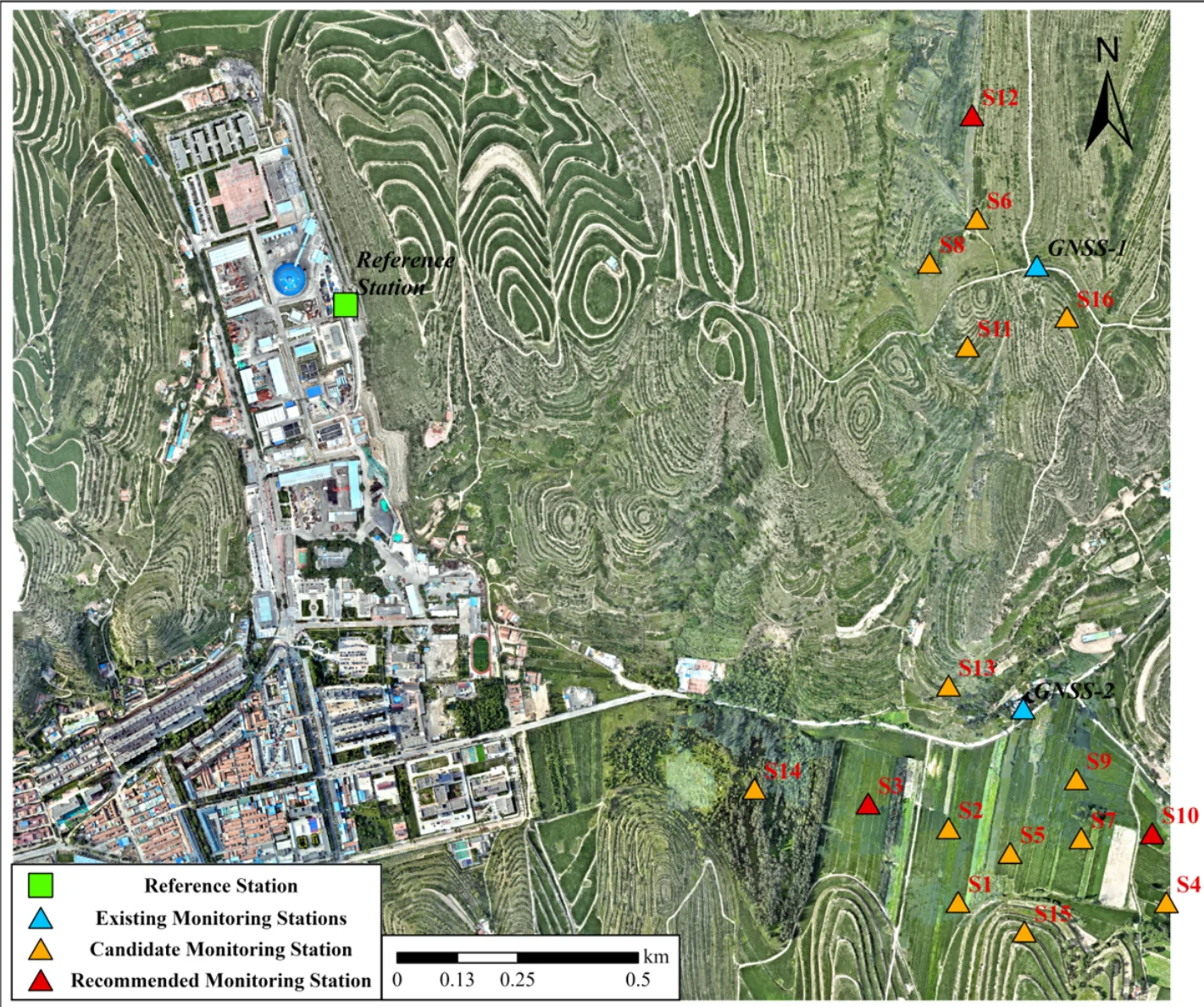
Spatial distribution of existing GNSS stations, candidate points, and final selected stations.

To examine the preliminary consistency between the proposed site-selection results and subsequent GNSS-observed subsidence responses, two existing monitoring stations (GNSS-1 and GNSS-2 in Figure 13) were used for a small-sample comparison. Because the proposed method is designed for a short-baseline RTK differential monitoring mode, validation employed the three-dimensional displacement time series from this monitoring system to compare the actual subsidence responses of the two stations. For this short-term validation, the reference station (green block in Figure 13) was used as the reference for the RTK displacement series. However, no independent CORS-constrained or repeated static GNSS verification of the reference station was available during the two-month period. The resulting series should therefore be interpreted as short-term relative displacements subject to this reference-stability assumption. Its relative stability was assessed from the available InSAR deformation pattern, terrain interpretation, and field reconnaissance, which indicated that it was located outside the main subsidence-response zone. Therefore, the two-month GNSS displacement series were used for a preliminary consistency assessment. Cumulative subsidence was calculated from the vertical component. Following the empirical consistency-assessment strategy used by Xu et al. [12], in which GNSS site-suitability values were compared with subsequent GNSS displacement time series, GNSS-1 and GNSS-2 were used to examine whether the model-based site-priority result was consistent with later GNSS-measured subsidence behavior.

GNSS-1 and GNSS-2 were overlaid on the monitoring-station suitability map and the related evaluation-factor layers. The corresponding standardized factor values and suitability values were then extracted for each station (Table 3). The results show that GNSS-2 had higher values for both deformation-intensity and cumulative-subsidence compared to GNSS-1. The corresponding H-GMSSM values were 0.62 for GNSS-2 and 0.60 for GNSS-1. This indicates that GNSS-2 has a relatively stronger capacity to represent the subsidence response in the model evaluation.

**Figure 14:**
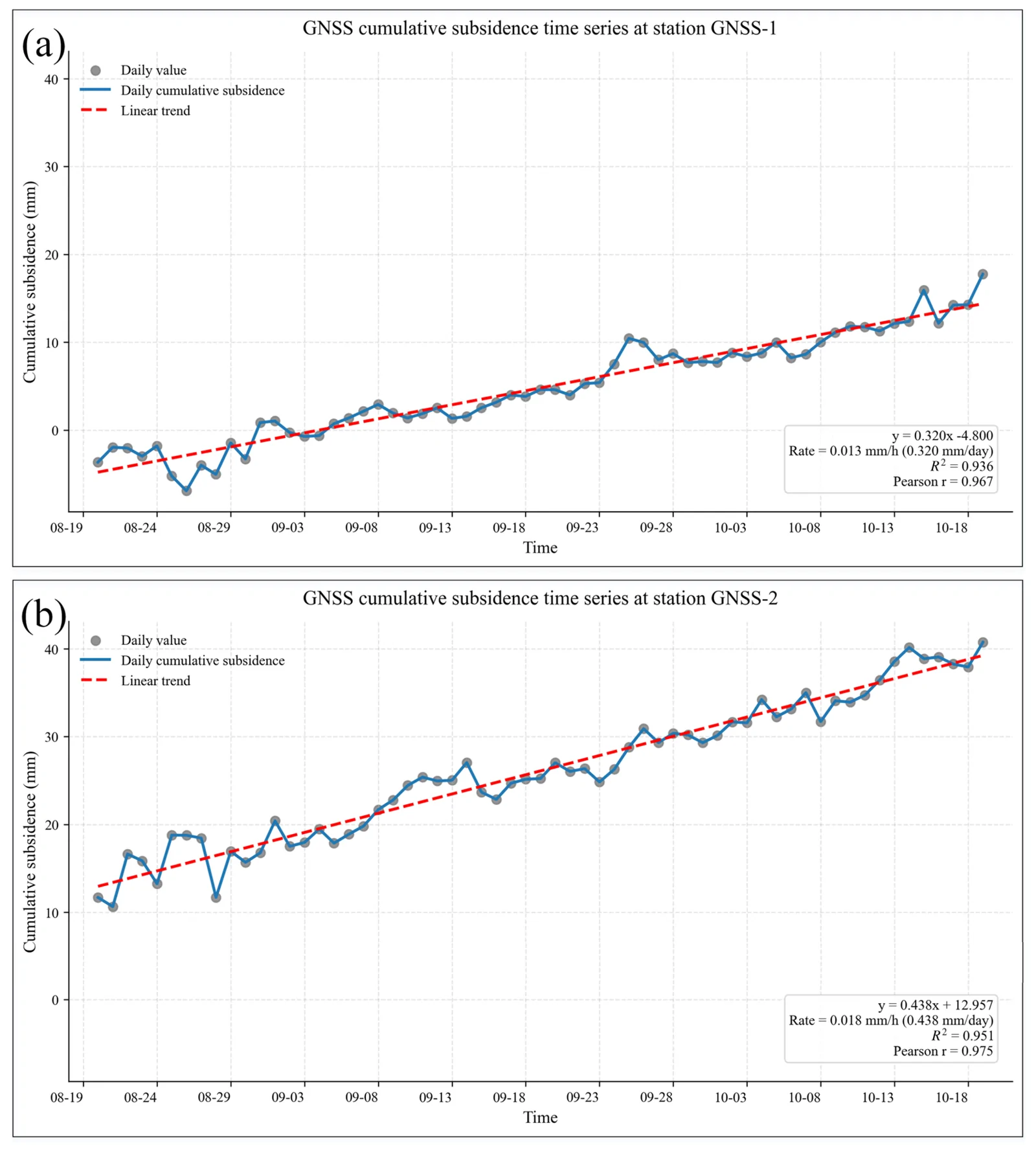
Cumulative-subsidence time series and linear fitting results for GNSS-1 and GNSS-2.

Furthermore, cumulative subsidence from 20 August to 20 October 2025 was calculated from the elevation time series of GNSS-1 and GNSS-2, and linear fitting was performed to derive the subsidence rates. Figure 14 shows the cumulative-subsidence time series and fitting results for the two stations using identical horizontal and vertical axis ranges. The fitted subsidence rates of GNSS-1 and GNSS-2 are 0.320 mm day^−1^ and 0.438 mm day^−1^, respectively. Pearson correlation coefficients between cumulative subsidence and time are 0.967 and 0.975 for GNSS-1 and GNSS-2, respectively. GNSS-2 exhibited higher values for deformation-intensity and cumulative-subsidence, alongside a faster fitted subsidence rate. Given the limited number of existing GNSS stations available for validation and their spatial distribution constrained by historical deployment conditions, this small-sample assessment cannot statistically demonstrate the general effectiveness of the model. Nevertheless, the consistency between the suitability evaluation results and the measured GNSS subsidence responses provides preliminary support for the rationality of the proposed method.

## 5. Discussion

The main contribution of this study is the development of a network-oriented site-selection framework that converts multi-source remote-sensing data into deployable priorities for GNSS monitoring stations in underground coal-mining subsidence areas. Rather than selecting stations solely according to the largest measured deformation or the highest pixel-level suitability, the framework balances the need for deformation representativeness with the reality of incomplete InSAR observations, while accounting for terrain, accessibility, and the additional spatial contribution of each station to the existing short-baseline RTK network. Thus, InSAR data gaps within or adjacent to active subsidence zones are regarded as spatial indicators of supplementary ground-monitoring demand. By integrating AHP-based suitability assessment, constrained RF correction, grid aggregation, GMM screening, minimum-distance thinning, field reconnaissance, and network review, the method converts a continuous suitability surface into a deployable station configuration. This approach preserves deformation representativeness while improving spatial complementarity within the existing network.

Xu et al. [12] demonstrated that historical InSAR deformation, UAV-derived terrain information, and AHP can quantify GNSS site selection for landslide. This study adapts this concept to underground mining areas characterized by basin-shaped subsidence, steep deformation gradients, localized InSAR gaps, and an existing RTK monitoring network. Furthermore, previous mining studies have demonstrated the complementary roles of different monitoring techniques. For example, Hu et al. [5] showed that GNSS can supplement incomplete InSAR observations in large-gradient subsidence areas. Tang et al. [26] reported that abrupt mining subsidence with large displacement gradients may not be reliably recovered by InSAR time series because of phase aliasing, a limitation that high-temporal-resolution GPS observations can overcome. Similarly, Zhu et al. [27] demonstrated that UAV, D-InSAR, and SBAS offer distinct advantages in the central, moderate, and peripheral zones of a subsidence basin. Unlike these studies, which mainly compare or integrate observations after monitoring has already begun, our framework moves this complementarity to the network-planning stage and uses historical deformation and InSAR incompleteness to determine where additional GNSS monitoring capacity should be deployed. The introduction of the RF model extends conventional AHP-based suitability mapping. The RF model was not intended to reconstruct missing deformation or independently predict future subsidence, but to prevent potentially important data-sparse areas from being excluded solely because of incomplete InSAR observations. Although variables like NDVI, slope, and TRI cannot directly represent subsurface mining mechanisms and the RF model showed modest discrimination, this uncertainty was controlled by restricting the RF output to a low-weight auxiliary correction within InSAR data-sparse zones. Sensitivity analyses confirmed that the overall suitability pattern and recommended-station rankings remained generally stable across alternative RF settings, AHP weights, InSAR-coverage thresholds, distance parameters, grid scales, and GMM configurations (Supplementary Tables S1–S13). This demonstrates that the final recommendations were not controlled by the RF auxiliary layer or by any single empirical assumption.

Several limitations define the scope of these findings. First, the InSAR coverage ratio represents data availability rather than complete measurement reliability [28,29,30,31]. Additionally, mining-engineering and geological controls were not included because spatially consistent datasets were unavailable. Second, the comparison with two existing GNSS stations over two months provides preliminary consistency evidence rather than statistical validation or representation of a complete mining cycle. Finally, some parameters were specifically calibrated for the present compact, short-baseline network and will require local adjustment before being transferred to other mining environments.

Future research should address these limitations at the model, site, and network levels. At the model level, mining depth, extraction thickness, working-face geometry and advance, overburden properties, faults, and more physically based InSAR quality indicators should be incorporated to improve the physical interpretability of the regional suitability assessment. At the site level, the recommended locations should be further evaluated using the criteria emphasized by Kakoullis et al. [10], including sky visibility, multipath risk, local geological and monument stability, accessibility, and receiver-level signal quality. Horizon analysis, DOP simulation, interference assessment, trial GNSS observations, and independent checks of reference-station stability should therefore be completed before construction. At the network level, deploying additional spatially independent stations and longer, multi-season GNSS time series, together with newly acquired InSAR observations and mining-progress information, will enable prospective validation and periodic network review, thereby supporting additional station deployment or network optimization as operational needs evolve.

## 6. Conclusions

This study developed a multi-source remote-sensing-assisted framework for GNSS monitoring-station site selection in underground coal-mining subsidence areas. The framework integrates InSAR-derived deformation and coverage information, optical and UAV-derived surface conditions, engineering accessibility, AHP-based suitability assessment, constrained RF assistance in InSAR data-sparse zones, and network-oriented candidate screening.

The results show that optimal GNSS stations are not necessarily located at pixels with the largest measured deformation. Instead, high-suitability areas concentrate mainly along subsidence basin margins and near InSAR coverage gaps, effectively balancing deformation representativeness, supplementary monitoring demand, and engineering feasibility. Sixteen candidate stations were identified, and S12, S3, and S10 were finally recommended to improve the northern, southwestern, and southeastern coverage of the existing short-baseline RTK network, respectively. Sensitivity analyses confirmed that the overall suitability pattern and recommended-station rankings remained stable under the tested parameter variations. The station with the higher suitability score also exhibited the higher fitted GNSS subsidence rate, (0.438 vs. 0.320 mm day^−1^). Although constrained by the limited validation sample and short observation period, this agreement provides preliminary consistency evidence rather than statistical validation.

Overall, the principal contribution of this study lies in transforming pixel-scale remote-sensing suitability information into network-oriented GNSS deployment priorities and in treating InSAR data gaps near active subsidence zones as spatial indicators of supplementary ground-monitoring demand. The framework can support more targeted field investigation and monitoring-network expansion in mining areas with incomplete InSAR observations. Its applicability is nevertheless limited by the small validation sample, the use of InSAR coverage as a proxy for data availability, the absence of detailed mining-engineering and geological variables, and the need for local parameter calibration. Future studies should incorporate more physically based InSAR quality indicators and mining-control information, strengthen validation through additional stations and longer GNSS time series, and recalibrate and evaluate the framework under different geological and network conditions.

## Figures and Tables

**Table 1:** Multi-source datasets for GNSS station site selection in the Pengyang mining subsidence area.

Dataset	Source	Spatial Resolution	Application
Sentinel-1A SAR imagery	Copernicus Programme, European Space Agency (ESA), Paris, France	5 m × 20 m	SBAS-InSAR-derived mean LOS deformation rate, cumulative subsidence, and effective coverage ratio
External DEM	OpenTopography, San Diego Supercomputer Center (SDSC), San Diego, United States	30 m	Topographic phase correction for InSAR time-series
DEM (UAV)	DJI Mavic 3E UAV survey data, SZ DJI Technology Co., Ltd. (DJI), Shenzhen, China	0.027 m	Derivation of slope and TRI from terrain-filtered elevation; terrain constraints for engineering-accessibility analysis
DOM (UAV)	DJI Mavic 3E UAV survey data, SZ DJI Technology Co., Ltd. (DJI), Shenzhen, China	0.027 m	Surface-background visualization, auxiliary interpretation of roads and ground objects, and presentation of final siting results
Sentinel-2C multispectral imagery	Copernicus Programme, European Space Agency (ESA), Paris, France	10 m × 10 m	NDVI-based vegetation cover assessment
Road vector data	OpenStreetMap, OpenStreetMap Foundation (OSMF), Cambridge, United Kingdom	/	Construction of the distance-to-road factor and accessibility quantification for construction, maintenance, and transportation

**Table 2:** Truncated Saaty fundamental scale (1–4) for AHP pairwise comparisons.

Scale Value	Definition
1	Factor i and factor j are of equal importance
2	Factor i is slightly more important than factor j
3	Factor i is moderately more important than factor j
4	Factor i is strongly more important than factor j
1/2, 1/3, 1/4	Reciprocals of above

Note: i denotes the row factor and j denotes the column factor in the judgment matrix.

**Table 3:** Normalized factor values and suitability values extracted for the two existing GNSS monitoring stations.

Station	NDVI	Slope	TRI	Deformation Intensity	Cumulative Subsidence	InSAR Coverage	DSE	Distance to Road	*P*_blind_	H-GMSSM
GNSS-1	0.62	0.94	0.92	0.88	0.88	0.12	0.11	0.50	0.41	0.60
GNSS-2	0.47	0.90	0.28	1.00	1.00	0.00	0.33	0.41	0.46	0.62

## Data Availability

The data are available from the corresponding author on reasonable request.

## Supplementary Materials

The following supporting information can be downloaded at: https://www.mdpi.com/article/10.3390/s26165057/s1, Table S1. RF threshold-sensitivity performance and feature importance; Table S2. Model comparison under the Q75 positive-label threshold; Table S3. Raster-pattern sensitivity analysis of the RF fusion weight; Table S4. Rank sensitivity of the recommended stations under different RF fusion weights; Table S5. Raster-level sensitivity analysis of the InSAR coverage-ratio threshold; Table S6. Rank sensitivity of the recommended stations under different InSAR coverage-ratio thresholds; Table S7. Raster-level sensitivity analysis of AHP weight perturbation; Table S8. Rank sensitivity of the recommended stations under AHP weight perturbation; Table S9. Sensitivity analysis of grid size and MAX/MEAN grid-score weighting; Table S10. Sensitivity analysis of the minimum-distance constraint; Table S11. Raster-level sensitivity analysis of DSE and road-distance Gaussian parameters; Table S12. Actual DSE and road-distance values of the recommended stations; Table S13. AIC/BIC comparison and final candidate-grid retention under different GMM component numbers.

## Abbreviations

The following abbreviations are used in this manuscript:

GNSS	Global Navigation Satellite System
InSAR	Interferometric synthetic aperture radar
RTK	Real-time kinematic
SAR	Synthetic aperture radar
SBAS-InSAR	Small baseline subset interferometric synthetic aperture radar
UAV	Unmanned aerial vehicle
DEM	Digital elevation model
DOM	Digital orthophoto map
NDVI	Normalized difference vegetation index
TRI	Terrain ruggedness index
DSE	Distance to subsidence edge
GIS	Geographic Information System
AHP	Analytic hierarchy process
RF	Random forest
OOB	Out-of-Bag
AUC	Area Under the Receiver Operating Characteristic Curve
GMM	Gaussian mixture model
AIC	Akaike Information Criterion
BIC	Bayesian Information Criterion
LOS	Line of sight
UTM	Universal Transverse Mercator
GMSSM	GNSS monitoring-station suitability map
H-GMSSM	Hybrid GNSS monitoring-station suitability map
CORS	Continuously operating reference stations
