# Supplementary material for: A Multi-Source Remote-Sensing-Assisted Method for GNSS Station Site Selection in Underground Coal-Mining Subsidence Areas
Source: Sensors (Basel). 2026 Aug 9;26(16):5057. doi: 10.3390/s26165057 (PMC13517356; doi:10.3390/s26165057)
Supplement: Supplementary file 1 [file sensors-26-05057-s001.zip › sensors-4370028-supplementary.pdf]

Supplementary Materials for "A Multi-Source Remote-Sensing-Assisted Method for GNSS Station Site Selection in Underground Coal-Mining Subsidence Areas"

Scope of sensitivity results. Each sensitivity table reports comparisons within its corresponding experiment. Table S13 alone reports the final posterior-probability-screened candidate-grid set obtained from the final comprehensive grid-score dataset.

Table S1. RF threshold-sensitivity performance and feature importance.

Threshold	Positive-label threshold (D)	Positive-sample ratio	AUC	Precision	Recall	F1-score	OOB error	TN	FP	FN	TP	NDVI importance	Slope importance	TRI importance
Q70	0.082156	0.300	0.592	0.389	0.325	0.354	0.361	4668	1309	1728	833	0.350	0.327	0.323
Q75	0.096502	0.250	0.573	0.325	0.230	0.269	0.312	5384	1019	1644	491	0.346	0.328	0.326
Q80	0.118948	0.200	0.569	0.268	0.163	0.203	0.257	6067	763	1429	279	0.349	0.326	0.326

Note. TN, FP, FN, and TP denote true negatives, false positives, false negatives, and true positives, respectively. TRI denotes the terrain ruggedness index input to the random forest (RF) model.

Table S2. Model comparison under the Q75 positive-label threshold.

Model	AUC	Precision	Recall	F1-score	FP	OOB error
RF	0.573	0.325	0.230	0.269	1019	0.312
LR	0.554	0.271	0.610	0.375	3511	—
AdaBoost	0.581	0.500	0.002	0.005	5	—

Model	AUC	Precision	Recall	F1-score	FP	OOB error
GBDT	0.590	0.467	0.007	0.013	16	—
XGBoost	0.595	0.444	0.004	0.007	10	—

Note. Out-of-bag (OOB) error was calculated only for the RF model as an internal evaluation metric. Although GBDT and XGBoost produced slightly higher AUC values than RF, their recall values were close to zero under the default probability threshold, indicating limited detection of positive samples. Logistic Regression achieved higher recall but generated substantially more false positives.

Table S3. Raster-pattern sensitivity analysis of the RF fusion weight.

RF weight	AHP weight	Pearson correlation with RF weight = 0.15 reference	Spearman correlation with RF weight = 0.15 reference	Top 10% overlap	Top 10% Jaccard
0.00	1.00	0.994	0.997	0.869	0.768
0.05	0.95	0.997	0.998	0.911	0.836
0.15	0.85	1.000	1.000	1.000	1.000
0.25	0.75	0.996	0.998	0.899	0.817

Note. The RF weight of 0.15 was used as the reference setting in this experiment. RF correction was applied only in areas with C_{InSAR} < 0.60. Pearson correlation, Spearman correlation, top-10% overlap, and the Jaccard index were calculated relative to the H-GMSSM raster obtained with RF weight = 0.15.

Table S4. Rank sensitivity of the recommended stations under different RF fusion weights.

Recommended station	Rank among the 16 fixed candidate stations			
	RF weight = 0.00	RF weight = 0.05	RF weight = 0.15	RF weight = 0.25
S3	5	5	5	5
S10	14	14	14	12
S12	9	9	9	9

Note. RF fusion weights of 0.00, 0.05, 0.15, and 0.25 were tested, with 0.15 used as the reference setting. Candidate identifiers and coordinates were fixed using the final 16-station candidate set. For each RF-weight scenario, H-GMSSM values were extracted at the same three station locations and ranked among the same 16 candidate stations. S3 and S12 retained identical ranks under all tested weights, while S10 varied slightly from rank 14 to rank 12 only when the RF weight was 0.25. Final station determination also

considered monitoring-network complementarity, field reconnaissance, and engineering feasibility.

Table S5. Raster-level sensitivity analysis of the InSAR coverage-ratio threshold.

InSAR coverage-ratio threshold	Pearson with T60 baseline	Spearman with T60 reference	Top 10% overlap	Top 10% Jaccard
0.40	0.968	0.968	0.920	0.852
0.50	0.976	0.974	0.935	0.878
0.60	1.000	1.000	1.000	1.000
0.70	0.938	0.938	0.904	0.825
0.80	0.818	0.822	0.810	0.681

Note. T60 denotes the 60% InSAR coverage-ratio threshold used as the reference setting. For each threshold, the RF model and H-GMSSM raster were recalculated using the threshold-specific training mask. Pearson correlation, Spearman correlation, top-10% overlap, and the Jaccard index were calculated relative to the T60 result. These results are intended for within-experiment comparison of the coverage threshold.

Table S6. Rank sensitivity of the recommended stations under different InSAR coverage-ratio thresholds.

Recommended station	40% threshold	50% threshold	60% threshold	70% threshold	80% threshold
	Rank among 16 fixed candidates	Rank among 16 fixed candidates	Rank among 16 fixed candidates	Rank among 16 fixed candidates	Rank among 16 fixed candidates
S3	8	8	8	6	8
S10	15	15	15	14	15
S12	6	6	7	7	6

Note. Candidate identifiers and coordinates were fixed using the final 16-station candidate set. For each InSAR coverage-ratio threshold, the RF model and H-GMSSM raster were recalculated, and values were extracted at the same station locations. Ranks were calculated among the same 16 candidate stations under each threshold scenario.

Table S7. Raster-level sensitivity analysis of AHP weight perturbation.

Perturbation scenario	Perturbation level	Perturbed factor or group	Weight multiplier	Pearson with reference scenario	Spearman with reference scenario	Top 10% overlap	Top 10% Jaccard
Reference	Reference	None	1.0	1.000	1.000	1.000	1.000
NDVI weight -20%	Individual factor	NDVI	0.8	1.000	1.000	0.985	0.970
NDVI weight +20%	Individual factor	NDVI	1.2	1.000	1.000	0.987	0.974
Slope weight -20%	Individual factor	Slope	0.8	1.000	1.000	0.985	0.971
Slope weight +20%	Individual factor	Slope	1.2	1.000	1.000	0.987	0.975
TRI weight -20%	Individual factor	TRI	0.8	1.000	1.000	0.998	0.995
TRI weight +20%	Individual factor	TRI	1.2	1.000	1.000	0.998	0.995
Deformation-intensity weight -20%	Individual factor	Deformation intensity	0.8	0.999	0.999	0.992	0.985
Deformation-intensity weight +20%	Individual factor	Deformation intensity	1.2	1.000	0.999	0.993	0.986
Cumulative-subsidence weight -20%	Individual factor	Cumulative subsidence	0.8	0.999	0.999	0.992	0.985
Cumulative-subsidence weight +20%	Individual factor	Cumulative subsidence	1.2	1.000	0.999	0.993	0.986
InSAR-coverage weight -20%	Individual factor	InSAR coverage	0.8	1.000	0.999	0.996	0.993
InSAR-coverage weight +20%	Individual factor	InSAR coverage	1.2	1.000	0.999	0.997	0.993
DSE weight -20%	Individual factor	DSE	0.8	1.000	0.999	0.942	0.891
DSE weight +20%	Individual factor	DSE	1.2	1.000	1.000	0.954	0.913
Distance-to-road weight -20%	Individual factor	Distance to road	0.8	0.999	0.998	0.949	0.902
Distance-to-road weight +20%	Individual factor	Distance to road	1.2	0.999	0.998	0.947	0.899

Perturbation scenario	Perturbation level	Perturbed factor or group	Weight multiplier	Pearson with reference scenario	Spearman with reference scenario	Top 10% overlap	Top 10% Jaccard
Deformation-factor group weight -20%	Factor group	Deformation-related factors	0.8	0.997	0.996	0.986	0.972
Deformation-factor group weight +20%	Factor group	Deformation-related factors	1.2	0.999	0.998	0.987	0.975
Terrain-factor group weight -20%	Factor group	Terrain factors	0.8	1.000	1.000	0.984	0.968
Terrain-factor group weight +20%	Factor group	Terrain factors	1.2	1.000	1.000	0.986	0.972
Engineering-factor group weight -20%	Factor group	Engineering-related factors	0.8	0.999	0.998	0.982	0.965
Engineering-factor group weight +20%	Factor group	Engineering-related factors	1.2	0.999	0.998	0.983	0.968
Surface-cover factor weight -20%	Factor group	Surface-cover factor	0.8	1.000	1.000	0.985	0.970
Surface-cover factor weight +20%	Factor group	Surface-cover factor	1.2	1.000	1.000	0.987	0.974
InSAR-availability factor weight -20%	Factor group	InSAR-availability factor	0.8	1.000	0.999	0.996	0.993
InSAR-availability factor weight +20%	Factor group	InSAR-availability factor	1.2	1.000	0.999	0.997	0.993

Note. Each individual factor weight and grouped factor weight was independently perturbed by ±20%, followed by proportional renormalization of the remaining weights. The final normalized and gap-compensated factor rasters were kept fixed, so this test isolates the influence of AHP weights. The grouped perturbations comprised deformation-related factors, terrain factors, engineering-related factors, the surface-cover factor, and the InSAR-availability factor. Although perturbing the deformation-related factor group produced the largest grid-level change, the raster-level Pearson and Spearman correlations remained high, consistent with the monitoring objective of mining-subsidence observation.

Table S8. Rank sensitivity of the recommended stations under AHP weight perturbation.

Recommended station	Reference rank among 16 fixed candidates	Best rank among 16 candidates	Worst rank among 16 candidates
S3	8	6	9
S10	14	13	14
S12	5	5	5

Note. This table isolates uncertainty associated with the AHP weights; RF-assisted fusion was not included in these candidate-level values. The reference values correspond to the unperturbed AHP weights within this experiment. Candidate identifiers and coordinates were fixed, and ranks were recalculated among the same 16 candidate stations under each perturbation scenario. The rank ranges were calculated across all single-factor and grouped-factor ±20% perturbations. Final station determination was not based solely on ranking, but also considered monitoring-network complementarity, redundancy relative to existing GNSS stations, field reconnaissance, and engineering feasibility.

Table S9. Sensitivity analysis of grid size and MAX/MEAN grid-score weighting.

Parameter	Tested setting	Reference setting	Valid grids	High-component grid area (m^2)	Spatial overlap with the reference high-component area	Area-based Jaccard index with the reference high-component area	Interpretation
Grid size	100 m	150 m	525	950000	0.485	0.485	Finer grid; more fragmented high-suitability patches
	150 m		238	1957500	1.000	1.000	Reference setting
	200 m		143	1280000	0.632	0.619	Coarser grid; stronger spatial aggregation
MAX/MEAN weight	0.5/0.5	0.7/0.3	238	–	0.989	0.977	More emphasis on mean grid suitability
	0.7/0.3		238	–	1.000	1.000	Reference setting
	0.9/0.1		238	–	0.989	0.966	More emphasis on local maximum suitability

Note. For the grid-size sensitivity test, spatial overlap was calculated as the intersection area divided by the reference high-component area, and the Jaccard index was calculated from the corresponding high-component areas. These metrics are area-based rather than grid-ID-based.

Table S10. Sensitivity analysis of the minimum-distance constraint.

Minimum distance (m)	Input GMM-derived candidates	Kept candidates	Recommended stations retained	Interpretation
80	34	19	S3, S10, S12 retained	More candidates retained; higher redundancy
100	34	17	S3, S10, S12 retained	Slightly weaker thinning than baseline
120	34	16	S3, S10, S12 retained	Baseline setting
150	34	12	S3 and S12 retained; S10 not directly retained	Stronger thinning; potential complementary sites may be removed
200	34	11	S3 and S12 retained; S10 not directly retained	Overly strict thinning; candidate set becomes too sparse

Note. The 34 input candidates were the final GMM-derived candidate grids obtained using the three-component model and posterior probability threshold of 0.60 reported in Table S13. The 120 m minimum-distance threshold was used as the baseline. Candidate thinning was applied to the GMM-derived candidate set before final network-level review. Under 150 and 200 m thresholds, S10 was not directly retained by the thinning procedure, although a nearby retained candidate was located approximately 143.18 m from S10. Final station determination was not based solely on this distance threshold; monitoring-network complementarity, redundancy relative to existing GNSS stations, field reconnaissance, and construction feasibility were also considered.

Table S11. Raster-level sensitivity analysis of DSE and road-distance Gaussian parameters.

Perturbation scenario	Perturbed parameter	DSE preferred distance μ_{DSE} (m)	DSE decay width σ_{DSE} (m)	Distance-to-road preferred distance μ_{road} (m)	Distance-to-road tolerance width σ_{road} (m)	Pearson with reference scenario	Spearman with reference scenario	Top 10% overlap	Top 10% Jaccard
Reference	None	120	60	120	90	1.000	1.000	1.000	1.000
DSE preferred distance = 80 m	μ_{DSE}	80	60	120	90	0.974	0.946	0.615	0.444
DSE preferred distance = 160 m	μ_{DSE}	160	60	120	90	0.986	0.972	0.719	0.561
DSE decay width = 40 m	σ_{DSE}	120	40	120	90	0.997	0.996	0.924	0.859

Perturbation scenario	Perturbed parameter	DSE preferred distance μ_{DSE} (m)	DSE decay width σ_{DSE} (m)	Distance-to-road preferred distance μ_{road} (m)	Distance-to-road tolerance width σ_{road} (m)	Pearson with reference scenario	Spearman with reference scenario	Top 10% overlap	Top 10% Jaccard
DSE decay width = 80 m	σ_{DSE}	120	80	120	90	0.998	0.996	0.912	0.839
Distance-to-road preferred distance = 80 m	μ_{road}	120	60	80	90	0.990	0.980	0.821	0.697
Distance-to-road preferred distance = 160 m	μ_{road}	120	60	160	90	0.992	0.988	0.843	0.729
Distance-to-road tolerance width = 60 m	σ_{road}	120	60	120	60	0.997	0.995	0.893	0.806
Distance-to-road tolerance width = 120 m	σ_{road}	120	60	120	120	0.999	0.997	0.933	0.875

Note. Each scenario perturbed one Gaussian parameter while keeping the other parameters and factor layers fixed. Pearson correlation, Spearman correlation, top-10% overlap, and the Jaccard index were calculated relative to the reference setting.

Table S12. Actual DSE and road-distance values of the recommended stations.

Recommended station	Actual DSE distance (m)	Difference from DSE optimum (m)	Reference DSE suitability	Actual road distance (m)	Reference road suitability	Interpretation
S10	118.6	-1.4	1.000	88.7	0.941	Very close to the theoretical DSE optimum; high integrated suitability
S3	149.4	29.4	0.887	104.7	0.986	Located within the high-suitability transition zone and retained after network-level review.

Recommended station	Actual DSE distance (m)	Difference from DSE optimum (m)	Reference DSE suitability	Actual road distance (m)	Reference road suitability	Interpretation
S12	198.0	78.0	0.430	118.1	1.000	Farther from the DSE optimum, but retained because of network complementarity and strong road accessibility

Note. The theoretical optimum of the DSE Gaussian factor was 120 m. The reference DSE and road-distance suitability values were calculated using the Gaussian parameters reported in Table S11. Final station selection was not based solely on either distance factor, but also considered integrated suitability, monitoring-network complementarity, field reconnaissance, accessibility, and construction feasibility.

Table S13. AIC/BIC comparison and final candidate-grid retention under different GMM component numbers.

Number of components	AIC	BIC	Highest-component mean	Retained high-suitability grids	Retention ratio	Interpretation
1	-285.625	-278.680	0.506	238	1.000	No suitability partition; all grids retained
2	-384.270	-366.908	0.629	107	0.450	Lowest AIC/BIC, but high group is too broad for operational screening
3	-380.855	-353.077	0.716	34	0.143	Low-medium-high partition; retained for candidate-grid screening
4	-380.826	-342.631	0.722	34	0.143	Similar retained high-grid set but more fragmented partition
5	-377.298	-328.686	0.719	37	0.155	Increased complexity with limited screening benefit
6	-374.778	-315.750	0.719	37	0.155	Potential over-partitioning with limited additional benefit

Note. GMMs with one to six components were fitted to the comprehensive candidate-grid suitability scores. A grid was retained when it was assigned to the highest-mean component and its posterior probability for that component was at least 0.60. Although the two-component model produced the lowest AIC and BIC values, it retained 107 high-suitability grids, accounting for 44.96% of all valid grids, which was too broad for extracting a limited set of GNSS candidate grids. The three-component GMM was therefore retained because it provided an interpretable low-, medium-, and high-suitability partition and isolated a limited high-suitability tail for operational candidate-grid screening. For the three-component GMM, the component means were 0.400, 0.585, and 0.716.
